# Numb Promotes Cell Proliferation and Correlates with Poor Prognosis in Hepatocellular Carcinoma

**DOI:** 10.1371/journal.pone.0095849

**Published:** 2014-04-25

**Authors:** Jian Wu, Shun-Li Shen, Bin Chen, Jing Nie, Bao-Gang Peng

**Affiliations:** 1 Department of Hepatobiliary Surgery, The First Affiliated Hospital of Sun Yat-sen University, Guangzhou, Guangdong Province, PR China; 2 Department of Nephrology, Nanfang Hospital, Southern Medical University, Guangzhou, Guangdong Province, PR China; Xiangya Hospital of Central South University, China

## Abstract

**Background:**

Numb is an evolutionary conserved protein that plays critical roles in cell fate determination, cell adhesion, cell migration and a number of signaling pathways, but evidence for a substantial involvement of Numb in HCC has remained unclear. The present study was aimed to investigate the clinical and prognostic significance of Numb and its role in hepatocellular carcinoma (HCC).

**Methodology:**

The expression of Numb was detected in 107 cases of clinical paraffin-embedded hepatocellular carcinoma tissues,5 matched paris of fresh tissues and six hepatocellular cell lines by immunohistochemistry with clinicopathological analyses,RT-PCR or Western blot. Moreover, loss of function and gain of function assays were performed to evaluate the effect of Numb on cell proliferation in vitro.

**Conclusions:**

We found that Numb was obviously up-regulated in HCC tissues and cell lines (p<0.05). The Numb up-regulation correlated significantly with poor prognosis, and Numb status was identified as an independent prognostic factor. Over-expression of Numb increased proliferation in SMMC-7721 and BEL-7402 cells, while knock-down of Numb showed the opposite effect. Our study indicates that Numb up-regulation significantly correlates with cell proliferation and poor prognosis in hepatocellular carcinoma patients. It may be a useful biomarker for therapeutic strategy in hepatocellular carcinoma treatment.

## Introduction

Hepatocellular carcinoma (HCC) is the fifth most common cancer in the world and represents the third leading cause of cancer mortality worldwide, with a only 30% to 40% five-year postoperative survival rate [1].Although survival of patients with HCC has improved due to advances in surgical techniques and perioperative management, long-term survival after surgical resection remains low due to the high rate of recurrence and metastasis [2], [3], [4].Major risk factors for HCC include both environmental factors (such as infection with HBV and alcoholic liver diseases) and genetic/epigenetic alterations [5], [6]. However, the molecular mechanism of its development and progression remains largely unknown. Therefore, it is critical to understand the etiology and to illustrate the mechanisms underlying HCC initiation and progression, and further to indentify valuable factors for prognosis prediction and novel therapeutic strategies.

Numb is an evolutionary conserved protein that plays critical roles in cell fate determination. Mammalian Numb displays a higher degree of structural complexity and encodes four alternatively spliced transcripts generating four proteins, ranging from 65 to 72 kDa. Accordingly, Numb proteins display a complex pattern of functions such as the control of asymmetric cell division and cell fate choice, and interacting with Itch to ubiquitinate Notch and Gli1, with Mdm2 to hamper ubiquitination of p53, with endocytic machinery to control Notch trafficking and with cadherin and integrins to control cell adhesion and migration [7], [8], [9]. Besides the functions in the cellular and organelle membranes, Numb also participated in development and progression of human cancer. Recently, deregulation of Numb was found in breast cancer, lung cancer and brain tumor medulloblastoma [10], [11], [12]. Loss of Numb expression is relevant to the homeostasis of the normal mammary parenchyma and its subversion contributes to human mammary carcinogenesis.These findings suggest that Numb plays a dominant positive role in the development and progression of those cancers [12], [13]. However, whether Numb deregulation also occurs in human HCC remains unclear. To address this question, we sought to investigate the expression of Numb in HCC and evaluated its prognostic significance and role in HCC.

## Materials and Methods

### 1. HCC patient samples and cell lines

A retrospective cohort of 107 HCC patients who underwent hepatectomy at the First Affiliated Hospital of Sun Yat-Sen University, with a median follow-up time of 17 months, were included in this study. All patients were diagnosed with primary HCC, and none had received prior radiotherapy or chemotherapy before the surgery. The study was carried out with prior approval of the Committees for Ethical Review of Research involving Human Subjects of the First Affiliated Hospital of Sun Yat-Sen University (Guangzhou, China),and written informed consent on the use of clinical specimens for medical research was obtained from each patient or the next of kin, caretakers, or guardians on the behalf of the participants involved in our study. Of the 107 surgical operated HCC patients (100 men and 7 women, aged from 8 to 73 years old, mean age is 52 years old), 92 patients was serum HBV-positive, Elevated alpha-feto- protein (AFP; ≥200 ng/mL) was detected in 77 cases (72%). Liver cirrhosis was found in 60 patients (56%) (Table 1).

**Table 1:** Clinicopathologic features and the expression of Numb in 107 HCC patients.

	High Numb Group	Low Numb Group
Number	43	64
Age	40.49+13.43	47.12+14.45
Male	39	61
Female	4	3
HBsAg(+)	36	56
AFP(+)	32	45
Multiple tumor	17	20
Biliary Invasion	3	4
Lymph node Invasion	1	3
Vessel Invasion	11	22
N0 Caspsulation	15	17
Diameter(cm)	7.75+4.13	8.57+5.14
Edmonson		
I	0	3
II	34	46
III	9	15
Cirrhosis	23	37

The Sk-HEP-1, HepG2, Hep3B,PLC/PRF/5,SMMC-7721 and BEL-7402, and an immortalized normal human liver cell line, Chang liver, were obtained from the Cell Bank, Chinese Academy of Medical Sciences (Shanghai, China). The cells were maintained in high-glucose Dulbecco's modified Eagle's media (DMEM) (Gibco, Australia) supplemented with 10% fetal bovine serum (Gibco, Australia).

### 2. Plasmid constructs and transfection

The full-length Numb cDNA was amplified and cloned into the pReciever M68 expression vector (FulenGen, Guangzhou, China). The expression plasmids were transfected into cells using Lipofectamine 2000 (Invitrogen, Carlsbad, CA, USA) according to the manufacturer's instructions.

### 3. siRNA Transfection

Oligonucleotide siRNA duplex was synthesized by Shanghai Gene Pharma (Shanghai, China). siRNA sequences were as follows: Numb, 5′-GGACCTCATAGTTGACCAG-3′.The transfection of siRNA in SMMC-7721 and BEL-7402 cells was carried out with Lipofectamine 2000 (Invitrogen), according to the manufacturer's instructions.

### 4. RNA extraction and reverse transcription-PCR

Total RNA from cells and primary tumor samples was extracted using the Trizol reagent (Invitrogen) according to the manufacturer's instruction. The extracted RNA was pretreated with RNase-free DNase, and 2 Ag RNA from each sample was used for cDNA synthesis primed with random hexamers. For PCR-mediated amplification of Numb cDNA, an initial amplification using Numb-specific primers was done with a denaturation step at 95°C for 10 min followed by 30 denaturation cycles at 95°C for 60 s, primer annealing at 55°C for 30 s, and primer extension at 72°C for 30 s. On completion of the cycling steps, a final extension at 72°C for 5 min was carried out before the reaction was stopped and stored at 4°C. Real-time PCR was then employed to determine the fold increase of Numb mRNA in each of the primary tumors relative to the paired noncancerous tissues, with each pair taken from the same patient. Expression data were normalized to the geometric mean of the housekeeping GAPDH gene to control the variability in expression levels. Reverse transcription- PCR and real-time PCR primers were designed using the Primer Express Software version 2.0 (Applied Biosystems). Primer sequences are: Sense,5'-CAGTGCCCGAGGTGGAAGGA-3';

Antisense,5'- AGTGGTGCCATCACGACATATG-3'.

### 5. Analysis of Cell Cycle

1×10^6^ cells were harvested and washed in PBS, then fixed in 75% alcohol for 30 min at 4°C. After washing in cold PBS three times, cells were resuspended in 1 ml of PBS solution with 40 ug of propidium iodide (Sigma) and 100 ug of RNase A (Sigma) for 30 min at 37°C. Samples were then analyzed for their DNA content by FACSCalibur (Becton Dickinson, Mountain View, CA). The proliferative index (PIx) is a measure of the number of cells in a tumor that are dividing (proliferating). PIx = (S+G2M)/(G0/G1+S+G2M)?×100%.

### 6. Cell proliferation and colony formation assays

Cell proliferation was determined using BrdU incorporation analysis. Ten micrograms per milliliter of BrdU were added to the culture medium for 24 h. The cells were fixed with 100% ethanol for 10 min, incubated with 2 m HCL for 45 min and 0.1 m sodium tetraborate for 15 min at room temperature. Then the cells were incubated with a mouse monoclonal anti-BrdU antibody overnight at 4°C. and were incubated with fluorescein isothiocyanate-conjugated goat antimouse IgG for 1 h at room temperature. Hoechst 33342 was used to label nuclei. For colony formation assays, 500 cells were plated onto 6-well plates and incubated at 37°C, When the cells grew to visible colonies, the colonies were washed once with PBS and fixed with 4% paraformaldehyde for 20 min. Next, cells were then stained with crystal violet, and the numbers of colonies per well were counted.

### 7. Western Blot Analysis

Tissues or harvested cultured cells were homogenized in lysis buffer (50 mmol/L Hepes pH 7.5, 150 mmol/L NaCl, 10% glycerol, 1% Triton X-100, 1.5 mmol/L MgCl_2_, 1 mmol/L EGTA, 10 mmol/L NaF, 10 mmol/L Na_4_P_2_O_7_, 1 mmol/L Na_3_VO_5_, 1 mmol/L phenylmethylsulfonyl fluoride, 10 µg/ml leupeptin and 20 µg/ml aprotinin). Protein was quantified by the Bradford assay (Bio-Rad, Hercules, CA), equal amount of protein were separated on SDS-polyacrylamide gels and transferred onto nitrocellulose membranes (Amersham Biosciences, Piscataway, NJ). After blocking in 5% skim milk for 1 h at room temperature, membranes were incubated with indicated primary antibody at 4°C overnight followed by horseradish peroxidaseconjugated second antibody and detected by chemiluminescence (Amersham Biosciences, Piscataway, NJ). Quantification of the Western blot data were performed by measuring the intensity of the hybridization signals using the Image analysis program (Fluor-ChemTM 8900, Alpha Inotech).

### 8. Immunocytochemistry

The sections were deparaffinized and rehydrated, and endogenous peroxidase was inhibited with 0.3% H_2_O_2_ methanol. For antigen retrieval, slides were boiled in 0.01 M, pH 6.0 sodium citrate buffer for 15 min in a microwave oven. After blocked with the 5% normal goat serum, primary anti-Numb monoclonal antibody (Abcam, 1∶200) in blocking buffer (1∶50) was applied and the slides were incubated at 4 uC overnight. After incubation with secondary antibody, the visualization signal was developed with DAB. The degree of immunostaining was reviewed and scored independently by two observers based on the proportion of positively stained tumor cells and intensity of staining. Tumor cell proportion was scored as follows: 0 (no positive tumor cells), 1 (<35% positive tumor cells), 2 (35–70% positive tumor cells), and 3(>70% positive tumor cells). Staining intensity was graded according to the following criteria: 0 (no staining), 1 (weak staining  =  light yellow), 2 (moderate staining  =  yellow brown), and 3(strong staining  =  brown). Staining index was calculated as the product of staining intensity score and the proportion of positive tumor cells. Using this method of assessment, we evaluated Numb expression by determining the staining index, with scores of 0, 1, 2, 3, 4, 6, or 9. The cutoff value for high and low expression level was chosen based on a measure of heterogeneity with the log-rank test statistical analysis with respect to overall survival. An optimal cutoff value was identified: a staining index score of >5 was used to define tumors with high Numb expression and a staining index score of <5 was used to indicate low Numb expression.

### 9. Statistic analysis

Statistical differences between two groups were determined by the Student's t test. A *P*<0.05 was considered statistically significant. The results were expressed as mean±SD from at least three experiments.

## Results

### Numb is up-regulated in HCC

**Figure 1:**
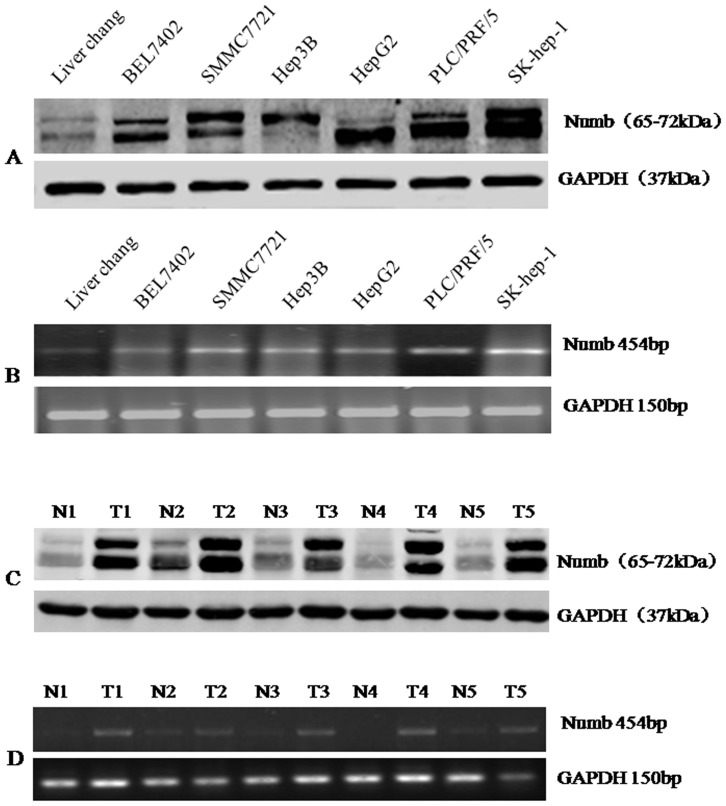
Numb is up-regulated in HCC cells and tissues. (A) Expression of Numb in normal and hepatoma cell lines was detected by Western blot. The same membrane was re-blotted with an anti-GAPDH antibody to verify equal loading. (B)Expression of Numb mRNA in normal and hepatoma cell lines was detected by reverse transcription-PCR. GAPDH was used as loading control. (C) Total protein extracted from tumor tissue (T) or adjacent non-tumor tissue (N) was immunobloted with antibody against Numb. The same membrane was re-blotted with an anti-GAPDH antibody to verify equal loading. (D) Expression of Numb mRNA in each of the primary tumors (T) and noncancerous tissues (N) paired from the same patient by reverse transcription-PCR. GAPDH was used as loading control.

Western blotting analysis revealed an evidently higher level of Numb expression in all 6 HCC cell lines than in normal liver cell line Liver Chang (Figure 1A). To clarify whether Numb up-regulation was occurring at transcriptional level, additional RT-PCR analyses were performed. Figure 1B demonstrated that the mRNA level of Numb in all HCC cell lines was obviously higher than that in normal liver cell line. These results demonstrated that Numb expression was elevated at both the mRNA level and the protein levels in the HCC cancer cell lines(Figure 1A and 1B). Additionally, we noted that Numb expression was relatively higher in the highly invasive HCC cell line SK-hep-1 than that in other HCC cell lines.

**Figure 2:**
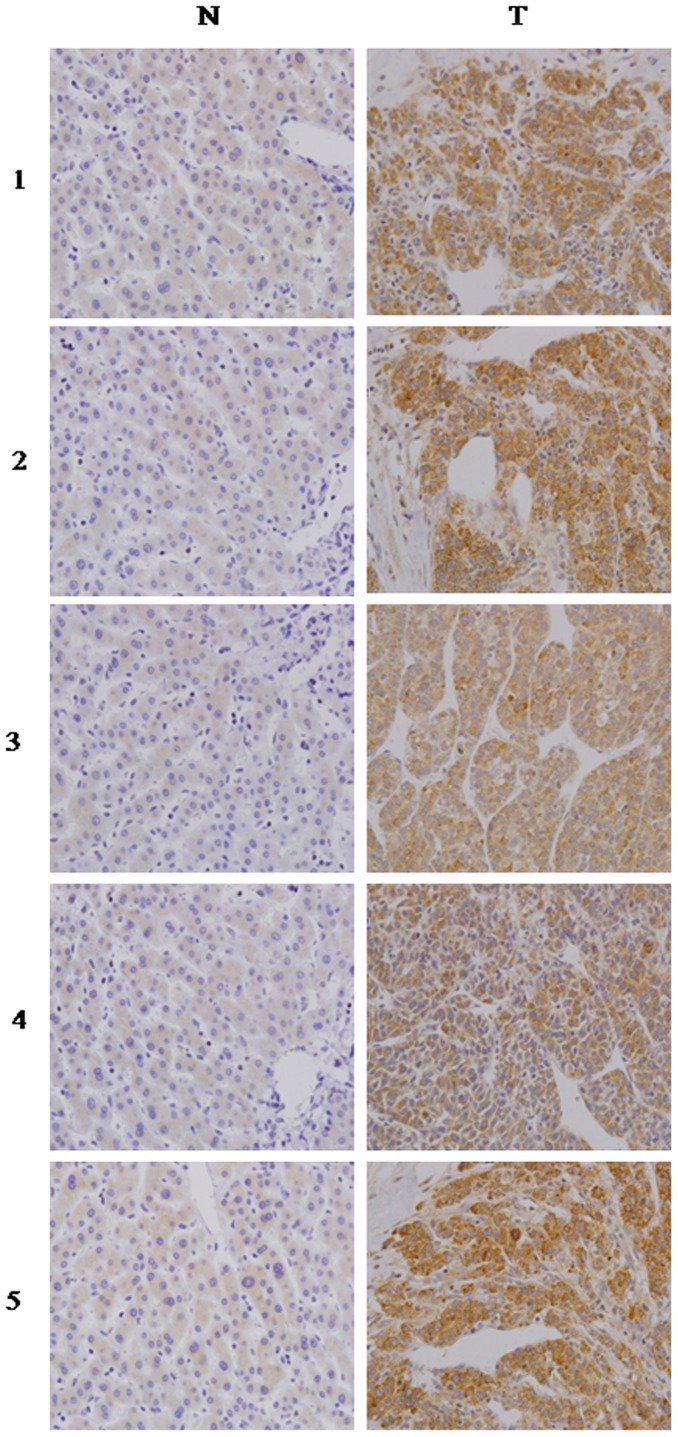
Numb expression level up-regulated in the primary HCC tissues compared with the paired adjacent non-cancerous tissues from the same patient, as examined by immunohistochemistry.

In order to determine whether the up-regulation of Numb in HCC cell lines is clinically correlated with HCC progression, we did Western blotting analysis and RT-PCR analysis on 5 pairs of matched normal liver tissue and HCC samples. As shown in Figure 2A and B, Numb was found to be differentially overexpressed in all 5 examined human primary HCC samples paired with normal liver tissues from the same patients. These findings are consistent with the results obtained in our immunohistochemical analysis (Figure 2C).

### High Numb expression is associated with poor prognosis of patients with HCC

**Figure 3:**
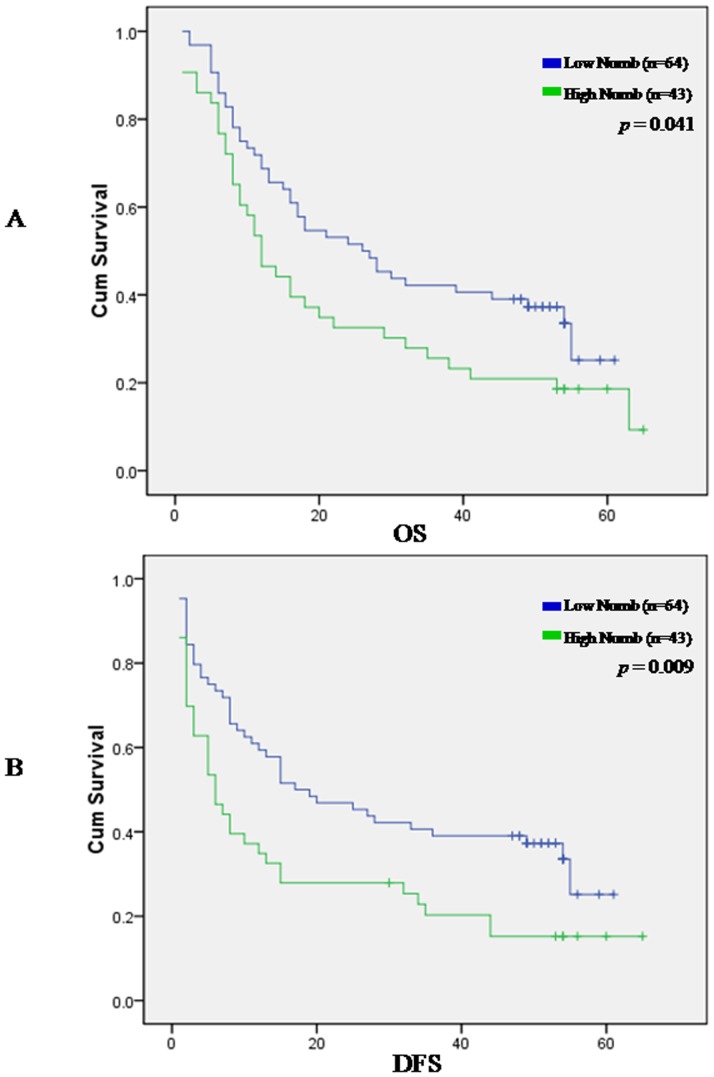
Kaplan-Meier survival curves for Numb expression in hepatocellular carcinoma (HCC) patients. The HCC patients with high Numb expression showed significantly shorter overall survival rates (*p* = 0.041, A) and disease-free survival (*p* = 0.009, B) than those with low Numb expression.

To determine the prevalence and clinical significance of Numb in liver cancer, we determined the expression of Numb protein by immunohistochemistry in a retrospective cohort of 107 pairs of tumor and matched adjacent nontumor tissue samples from HCC patients after liver resection. Numb immunoreactivity was graded according to the previously reported procedures. Cancer tissues from 96 of 107 (89.7%) patients revealed higher level of Numb protein expression in contrast to the expression of Numb in the adjacent noncancerous tissues. Only in 11 patients, level of Numb protein expression in adjacent noncancerous tissues is not less than in cancer tissues. Expression level of Numb (a staining index score of >5) was significantly elevated in tumor tissue samples compared with the nontumor counterparts (P<.0001). In the 107 cases examined, High Numb expression was detected in 43 (40.19%) HCC specimens, whereas only 22 (20.60%) of the nontumor specimens(Table 1).Next, clinical association analysis by the Pearson chi-square test revealed that Numb expression in HCC tumors was significantly associated with poor prognostic for HCC patients. For overall survival analysis, 107 HCC patients with sufficient and valid follow-up data were included, As shown in Figure 3A, patients with low Numb expression had longer overall survival time, whereas those with high Numb expression had shorter overall survival time (Figure 3A, log-rank, P = 0.001); the median OS times in the High Numb group (n = 43) and Low Numb group (n 64) of HCC patients were 23.3 months (95% CI, 16.6 months-30.0months) and 31.9 months (95% CI, 26.3 months-37.6 months), respectively. Kaplan-Meier analysis revealed the association of Numb expression with short OS times (P = .041) (Fig.3A).The 1-,3-and 5- year survival rate decreased from 72%,42%,31% in low Numb group to 53%,26%,18% in high Numb group. Then we examined whether Numb expression was associated with tumor recurrence and DFS of HCC. Of the 107 patients who received curative surgery for HCC and with sufficient followup data, the median DFS times in the High Numb group (n = 43) and Low Numb group (n = 64) of HCC patients were 18.3 months (95% CI, 11.4 months-25.2 months) and 29.2 months (95% CI, 23.1 months-35.3 months), respectively. Kaplan-Meier analysis also revealed the association of Numb expression with short DFS times (P = .009) (Fig.3B).The 1-,3-and 5- year DFS rate decreased from 61%,41%,31% in low Numb group to 37%,21%,15% in high Numb group.

Univariate Cox regression analyses revealed that higher level of Numb, age, Tumor size, Tumor multiplicity, Lymphatic invasion were all were worse predictors for overall survival of HCC patients (Table 2). In addition, multivariate Cox regression analysis revealed that higher level of Numb, age, Tumor size, Tumor multiplicity, Lymphatic invasion were independent prognostic markers for HCC (Table 3).

**Table 2:** Univariate analysis of different prognostic factors in 107 HCC patients by cox regression analysis

Variable	HR(95% CI)		p-value
	Lower	Upper	
Sex	.305	2.954	.928
Age	1.001	1.048	.038
Tumor multiplicity	.271	.862	.014
LN Invasion	.049	.586	.005
Biliary Invasion	.188	1.181	.109
Vessel Invasion	.427	1.638	.602
Caspsulation	.532	2.008	.923
Edmonson	.386	1.592	.501
Diameter	1.045	1.177	.001
Cirrhosis	.673	2.038	.576
HBsAg	.711	4.246	.225
AFP	1.000	1.000	.522
AFP_A	.334	1.590	.426
Numb	1.106	3.467	.021

**Table 3:** Multivariate analysis of different prognostic factors in 107 HCC patients by cox regression analysis.

Variable	HR(95% CI)		p-value
	Lower	Upper	
Age	1.003	1.040	.023
Tumor multiplicity	.315	.813	.005
LN Invasion	.078	.660	.006
Diameter	1.029	1.123	.001
Numb	1.128	2.912	.021

Taken together, these results indicate that Numb could be helpful to evaluate the prognosis in HCC patients.

### Correlation of Numb protein expression with clinicopathologic features in 107 HCC patients

We further examined the possible correlations between expression levels of Numb and clinical features of HCC. However, Spearman correlation analysis did not show significant associations between Numb expression and clinical features of HCC.

### Numb promotes cellular proliferation

**Figure 4:**
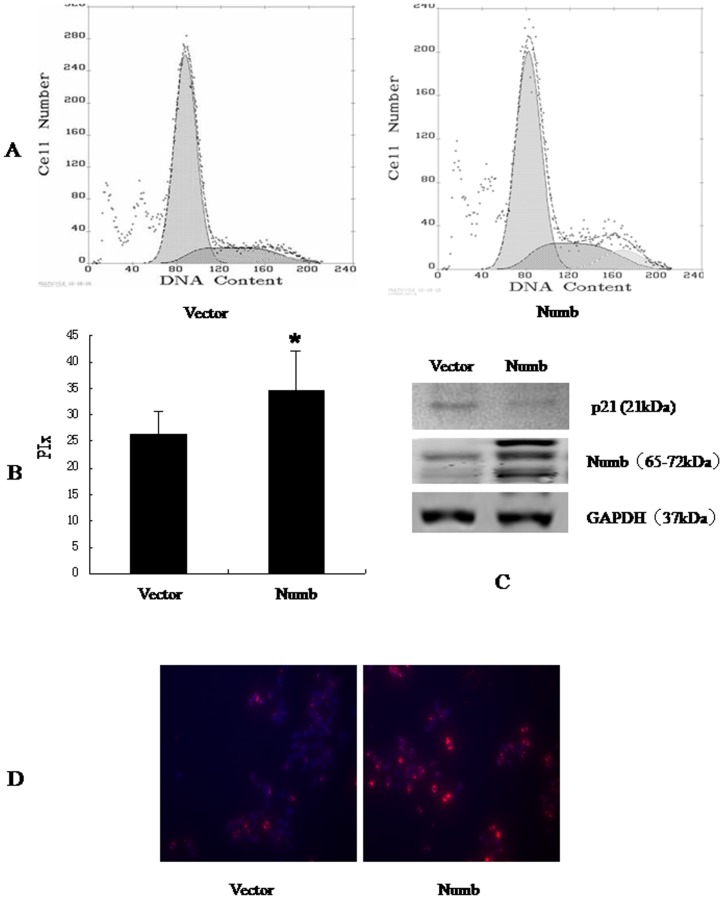
Overexpression of Numb down-regulated p21, promoted SMMC-7721 cell cycle progression. SMMC-7721 cells were transfected with empty vector, Ad-CMV-HA-Numb, respectively. 48 h after transfection, the amount of Numb and p21 was determined by antibodies specific for Numb and p21(C). Cell cycle was analysed by FACSCalibur (Becton Dickinson, Mountain View, CA) (A). The proliferative index (PIx) of Numb-overexpressed cell is higher than control (*P*<0.05, B). For BrdU incorporation analysis, the cells were incubated with a mouse monoclonal anti-BrdU antibody overnight at 4°C,and were incubated with fluorescein isothiocyanate-conjugated goat antimouse IgG for 1 h at room temperature. Hoechst 33342 was used to label nuclei (red). Photographs were taken using a Nikon microscope. Magnification100x. The proliferation cells (red) increased significantly.

**Figure 5:**
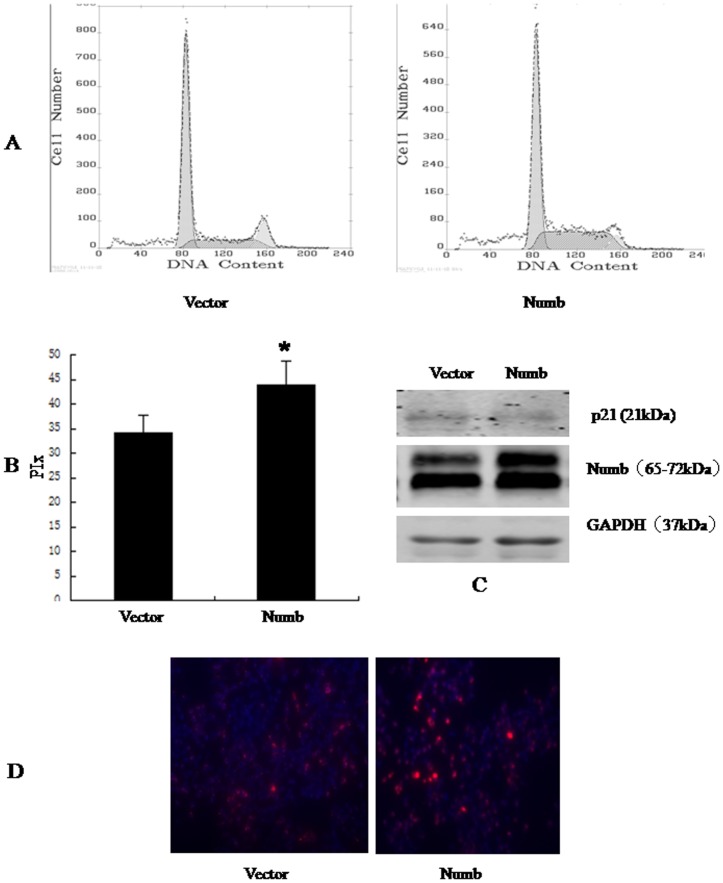
Overexpression of Numb down-regulated p21, promoted BEL-7402 cell cycle progression. BEL-7402 cells were transfected with empty vector, Ad-CMV-HA-Numb, respectively. 48 h after transfection, the amount of Numb and p21 was determined by antibodies specific for Numb and p21(C). (A)Cell cycle was analysed by FACSCalibur (Becton Dickinson, Mountain View, CA). The proliferative index (PIx) of Numb-overexpressed is higher than control (*P*<0.05, B). For BrdU incorporation analysis, the cells were incubated with a mouse monoclonal anti-BrdU antibody overnight at 4°C. and were incubated with fluorescein isothiocyanate-conjugated goat antimouse IgG for 1 h at room temperature. Hoechst 33342 was used to label nuclei (red). Photographs were taken using a Nikon microscope. Magnification100x. The proliferation cells (red) increased significantly.

**Figure 6:**
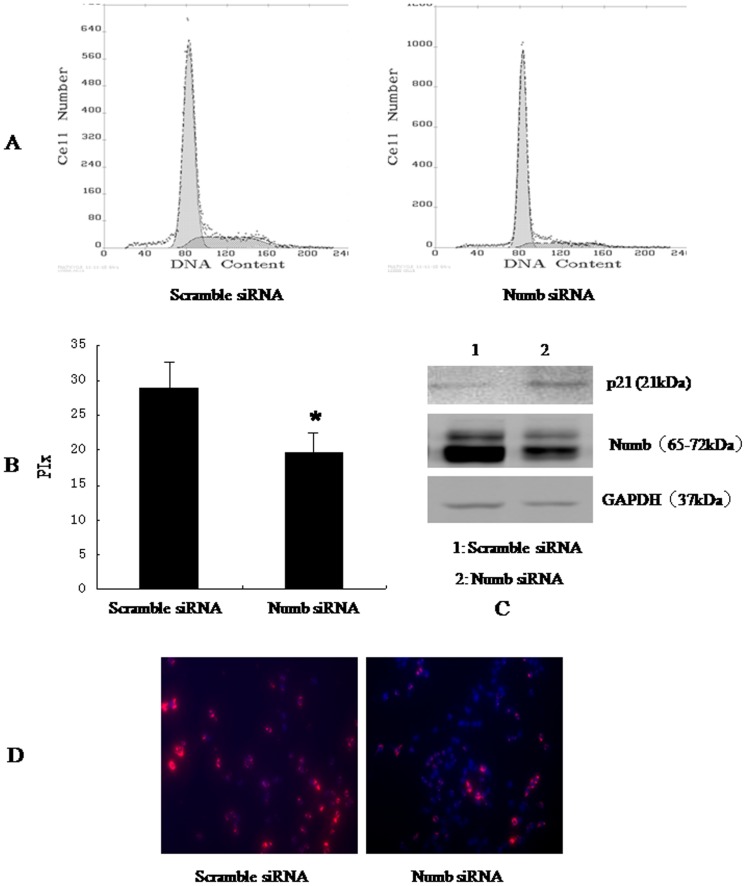
Konckdown of Numb up-regulated the expression of p21, inhibited SMMC-7721 cell cycle progression. SMMC-7721 cells were transfected with scramble siRNA, Numb siRNA, respectively. 48 h after transfection, the amount of Numb and p21 was determined by antibodies specific for Numb and p21(C). (A)Cell cycle was analysed by FACSCalibur (Becton Dickinson, Mountain View, CA). The proliferative index (PIx) decreased compared to control (*P*<0.05, B). For BrdU incorporation analysis, the cells were incubated with a mouse monoclonal anti-BrdU antibody overnight at 4°C, and were incubated with fluorescein isothiocyanate-conjugated goat antimouse IgG for 1 h at room temperature. Hoechst 33342 was used to label nuclei (red). Photographs were taken using a Nikon microscope. Magnification100x. The proliferation cells (red) decreased significantly.

**Figure 7:**
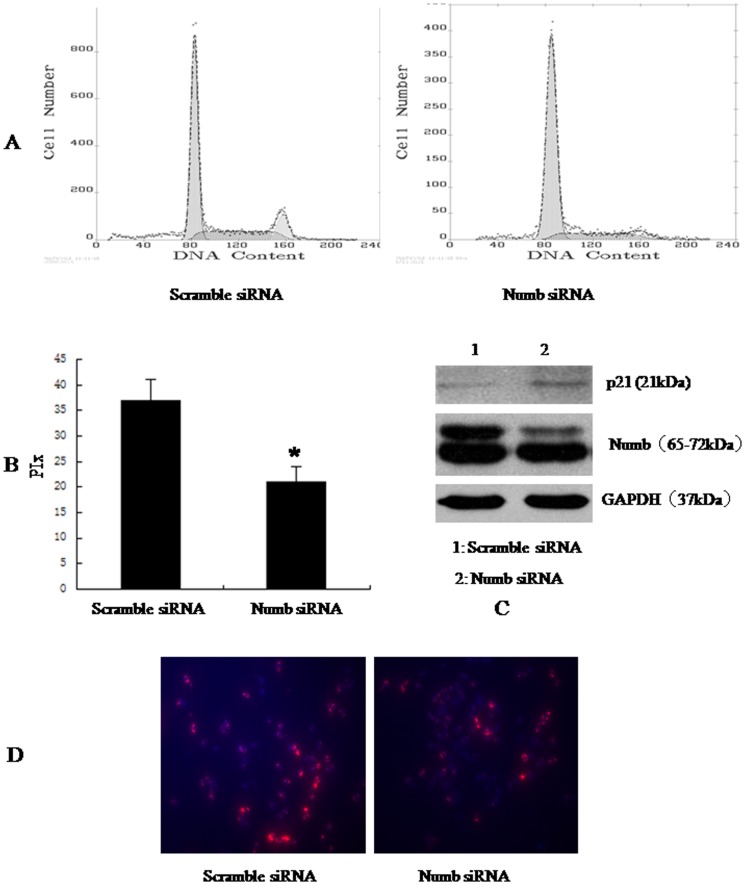
Konckdown of Numb up-regulated the expression of p21, inhibited BEL-7402 cell cycle progression. BEL-7402 cells were transfected with scramble siRNA, Numb siRNA, respectively. 48 h after transfection, the amount of Numb and p21 was determined by antibodies specific for Numb and p21(C). (A)Cell cycle was analysed by FACSCalibur (Becton Dickinson, Mountain View, CA). The proliferative index (PIx) decreased compared to control (*P*<0.05, B) For BrdU incorporation analysis, the cells were incubated with a mouse monoclonal anti-BrdU antibody overnight at 4°C,and were incubated with fluorescein isothiocyanate-conjugated goat antimouse IgG for 1 h at room temperature. Hoechst 33342 was used to label nuclei (red). Photographs were taken using a Nikon microscope. Magnification100x. The proliferation cells (red) decreased significantly.

**Figure 8:**
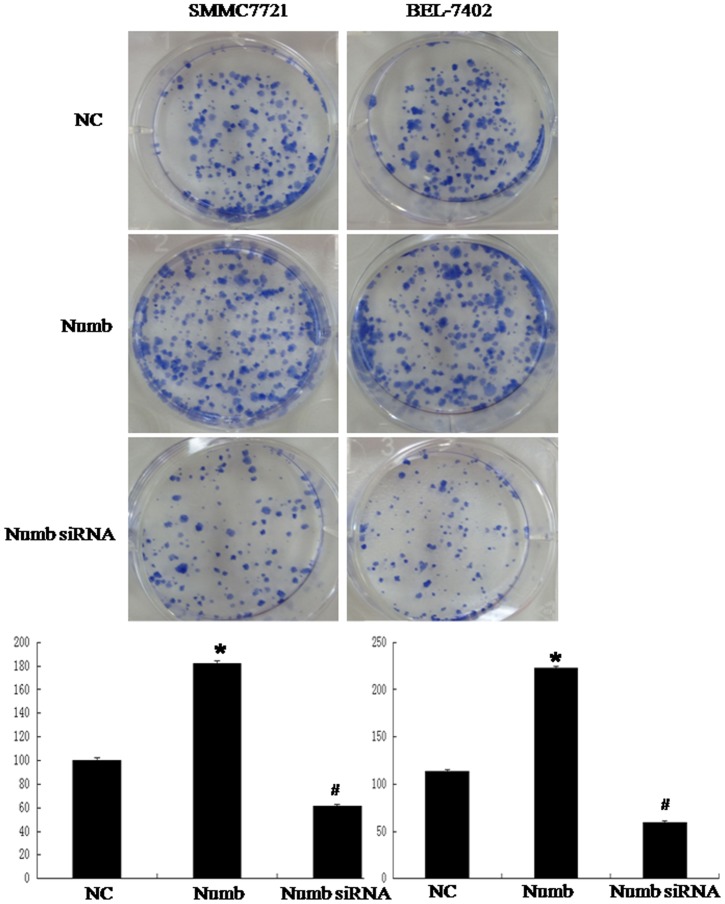
Overexpression of Numb in HCC cell lines promotes colony formation. (A) We cotransfected the Numb overexpression construct into SMMC-7721 and BEL-7402 cells. Overexpressed Numb promoted colony formation in SMMC-7721 and BEL-7402 cells. (P<0.05). Konckdown of Numb in SMMC-7721 and BEL-7402 cells showed the opposite effects (P<0.05). Representative pictures of colony formation assay as showed.

To better understand the role of Numb in HCC, we overexpressed Numb in SMMC-7721 and BEL-7402 cells. Stable Numb and control transfectants were generated and examined for Numb protein levels (Figure 4C and 5C).We analyzed cell cycle kinetics in SMMC7721 and BEL-7402 cells transfected with either Numb or control. We further analyses cell proliferation by BrdU incorporation analysis and Clone formation assay. Numb–overexpressing cells showed a statistically significant increase in proliferation when compared to Control cells, as assessed by Analysis of Cell Cycle and BrdU incorporation analysis (Figure 4,5 A,B and D). In order to confirm these findings, we next knocked down Numb levels in SMMC-7721 and BEL-7402 cells. Either specific-stranded RNA oligonucleotides against Numb or negative RNA control were transfected into SMMC-7721 and BEL-7402 cells.When Numb-specific oligonucleotides were used, a rapid downregulation of Numb was detected (Figure 6C and 7C). The decrease in Numb levels induces G0/G1 cell cycle arrest in SMMC7721 and BEL-7402 cells and result in a concomitant decrease in proliferation (Figure 6,7 A,B and D). Furthermore, Clone formation assay was performed (Figure 8). Overexpression of Numb increased the number of colonies, compared with control. Konckdown of Numb decreased the number of colonies, compared with control.

## Discussion

In this study, we first detected the expression of Numb in 107 cases of clinical paraffin-embedded HCC tissues, 6 HCC cell lines and 5 matched clinical fresh HCC tissues. IHC results showed that the positive signaling of Numb was observed in the cytoplasm of hepatocytes. Numb expression was significantly higher in HCC tissues than adjacent normal livers. We also found that Numb was up-regulated in four HCC cell lines as well as in 5 matched clinical fresh tissues. Our data also first clearly demonstrate the increased expression of Numb in HCC.

Then we examined the clinicopathological values of Numb in HCC tissues. Numb expression, age, Tumor size, Tumor multiplicity, Lymphatic invasion were independent prognostic factors relative to overall survival from multivariate analysis. Besides Numb expression, the four other factors are well-acknowledged indicators in the progression of HCC [14]. However, Spearman correlation analysis did not show significant associations between Numb expression and other clinical features, which may be related to mammalian Numb encoding four alternatively spliced transcripts generating four proteins, Numb1 (p72), Numb2 (p66), Numb3 (p71) and Numb4 (p65) isoforms.The correlation of different isforms expression with clinical features will be studied in future research. These data indicate that Numb expression is an independent prognostic marker for survival of HCC patients.

Recent evidence suggests an important role for Numb in modulating cancer progression. the ability of Numb to control oncogenic (Notch and Hedgehog) and tumor suppressor (p53) pathways provides a strong link between deregulated Numb function and cancer [8], [15], [16], [17]. Indeed, hyperactive Notch and Hedgehog functions have been described to be responsible of several tumors [18], [19], [20], [21]. Loss of Numb expression in breast cancer has been described to imbalance Notch (activation) and p53 (attenuation) oncogenic and tumor suppressor activities, respectively [12], [13]. Finally, loss of Numb expression in the Hedgehog-dependent brain tumor medulloblastoma has been reported to unrestrain Hedgehog activity and promote tumor growth [11].The complexity of Numb isoforms and multiple functions summarized here, suggests that these proteins may play several roles depending on distinct cell types and specific stages of development.

So we next explored the possible function of Numb in the progression of HCC. The results showed that forced expression of Numb in SMMC-7721 and BEL-7402 cells promote cell cycle progression in vitro. Depletion of Numb in SMMC-7721 and BEL-7402 showed the opposite effects. We also showed that Numb positively correlated with cell proliferation in SMMC-7721 and BEL-7402 cells by BrdU incorporation analysis and Clone formation assay.

In conclusion, this is the first study aimed at evaluating the role of Numb in HCC and possibility of using Numb as a prognostic marker for patient survival in HCC. Nevertheless, further investigation on the mechanism by which Numb is involved in the cell proliferation of HCC is required.
