# Correction: Numb Promotes Cell Proliferation and Correlates with Poor Prognosis in Hepatocellular Carcinoma

**DOI:** 10.1371/journal.pone.0265938

**Published:** 2022-03-23

**Authors:** Jian Wu, Shun-Li Shen, Bin Chen, Jing Nie, Bao-Gang Peng

After this article [1] was published, several issues came to light.

Specifically:

- In Fig 2, the lower portion of the ‘N’ panel for patient 2 appears similar to the upper portion of the ‘N’ panel for patient 3. The authors provided a corrected version of Fig 2 that includes replacement data for patient 2 from the original experiment. Underlying data for Fig 2 are in S1 File with this notice.
- Similarities were noted between the p21 blots in Fig 6C and Fig 7C, and between the data in lane 2 of these p21 panels and lane 1 of the p21 blot in Fig 4C. Fig 4 reports overexpression experiments conducted in SMMC-7721 cells, and Figs 6 and 7 report knockdown experiments conducted in SMMC-7721 and BEL-7402 cells, respectively. The authors provided raw image data to support these figure panels (S2, S3 and S4 Files) and commented that errors were made in preparing Figs 6 and 7 due to the large amount of data and similarities between the p21 blots for the three experiments. The raw images provided did not appear to match the published figure panels in some cases; the authors stated that some of the original data are no longer available, for those results they provided replicate data from the same set of experiments. Updated versions of Figs 6 and 7 are provided here. The authors stated that the p21, Numb, and GAPDH data reported in each updated figure are from the same internal experiment.
- Similarities were noted between the anti-BrdU immunofluorescence images shown in Fig 6D for SMMC-7721 cells and in Fig 7D for BEL-7402 cells. The authors commented that the SMMC-7721 data were erroneously reported in Fig 7D. In the updated version of Fig 7 provided here, the correct images from the original BEL-7402 experiment are provided in panels C and D. The underlying data for Fig 6 and the corrected Fig 7 are in S3 and S4 Files.
- The sense primer reported in [1] for Numb RT-PCR experiments does not appear to target NUMB. The authors noted that this reflects a reporting error and stated that the following primers were used in the NUMB RT-PCR reactions to amplify nucleotides 1602–2055 of the NM_001005745.1 transcript:
  ○ Sense 5’- TTCCTCACCTCTCAGCCTGT-3’
  ○ Antisense 5’-GGTTGGTAGGGGAGGGATTA-3’
- The Chang Liver cell line used in this study has been reported as misidentified or contaminated [2–4]. The authors confirmed that this line has been found to be indistinguishable from HeLa cells. They stated that this cell line was used only as a control in [1] and that the main experimental results were obtained in SMMC-7721 and MEL-7402 cells. However, due to the issue with Chang Liver cells, results and conclusions statements based on Fig 1A and B and which draw comparisons between Numb expression in HCC versus normal liver cell lines are not supported.
- The scramble sequence used as a control in siRNA experiments was not reported in the Materials and Methods. The sequence for this reagent is 5’-UUCUCCGAACGUGUCACGUTT-3’.
- Panel D was not cited in the legends for Figs 4, 5, 6, and 7. The text describing BrdU incorporation analysis refers to panel D for each of these figures. This has been updated in the Figs 6 and 7 legends provided with this notice.
- There are figure referencing errors in the second paragraph of the Results section in [1]. The corrected paragraph is: In order to determine whether the up-regulation of Numb in HCC cell lines is clinically correlated with HCC progression, we did Western blotting analysis and RT-PCR analysis on 5 pairs of matched normal liver tissue and HCC samples. As shown in Fig 1C and 1D, Numb was found to be differentially overexpressed in all 5 examined human primary HCC samples paired with normal liver tissues from the same patients. These findings are consistent with the results obtained in our immunohistochemical analysis (Fig 2).

**Fig 2:**
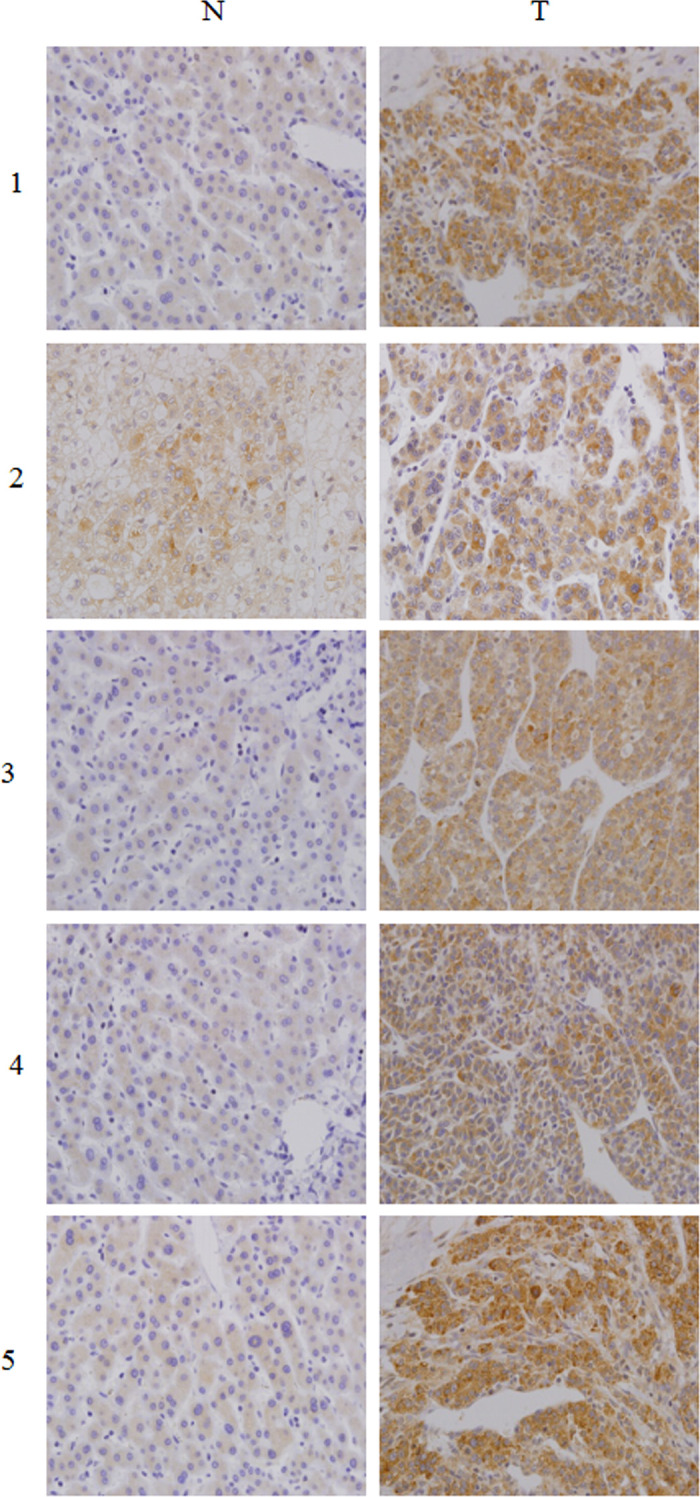
Numb expression level up-regulated in the primary HCC tissues compared with the paired adjacent non-cancerous tissues from the same patient, as examined by immunohistochemistry.

**Fig 6:**
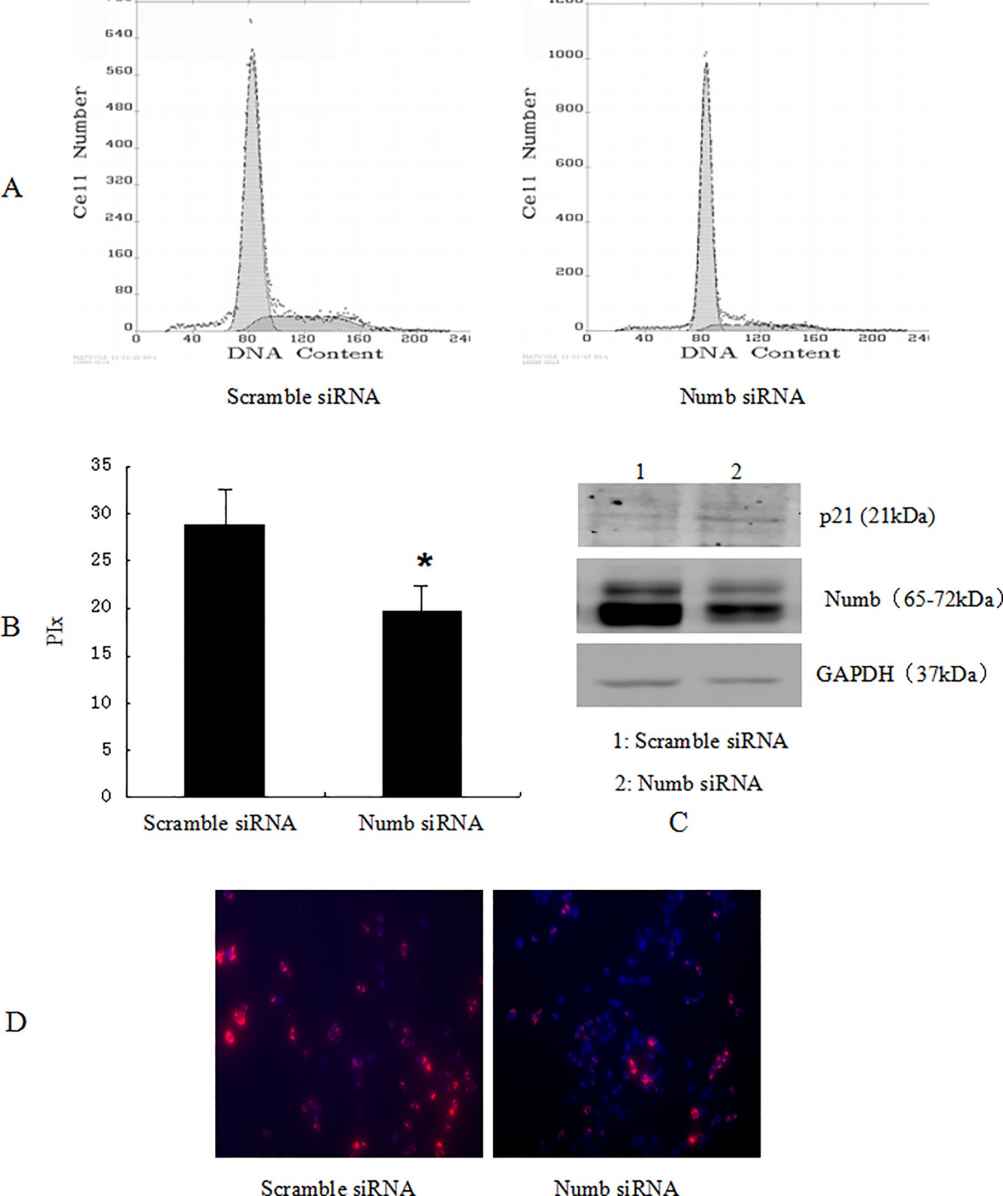
Knockdown of Numb up-regulated the expression of p21, inhibited SMMC-7721 cell cycle progression. SMMC-7721 cells were transfected with scramble siRNA, Numb siRNA, respectively. 48 h after transfection, the amount of Numb and p21 was determined by antibodies specific for Numb and p21(C). (A)Cell cycle was analysed by FACSCalibur (Becton Dickinson, Mountain View, CA). The proliferative index (PIx) decreased compared to control (P<0.05, B). For BrdU incorporation analysis (D), the cells were incubated with a mouse monoclonal anti-BrdU antibody overnight at 4°C, and were incubated with fluorescein isothiocyanate-conjugated goat antimouse IgG for 1 h at room temperature. Hoechst 33342 was used to label nuclei (red). Photographs were taken using a Nikon microscope. Magnification100x. The proliferation cells (red) decreased significantly.

**Fig 7:**
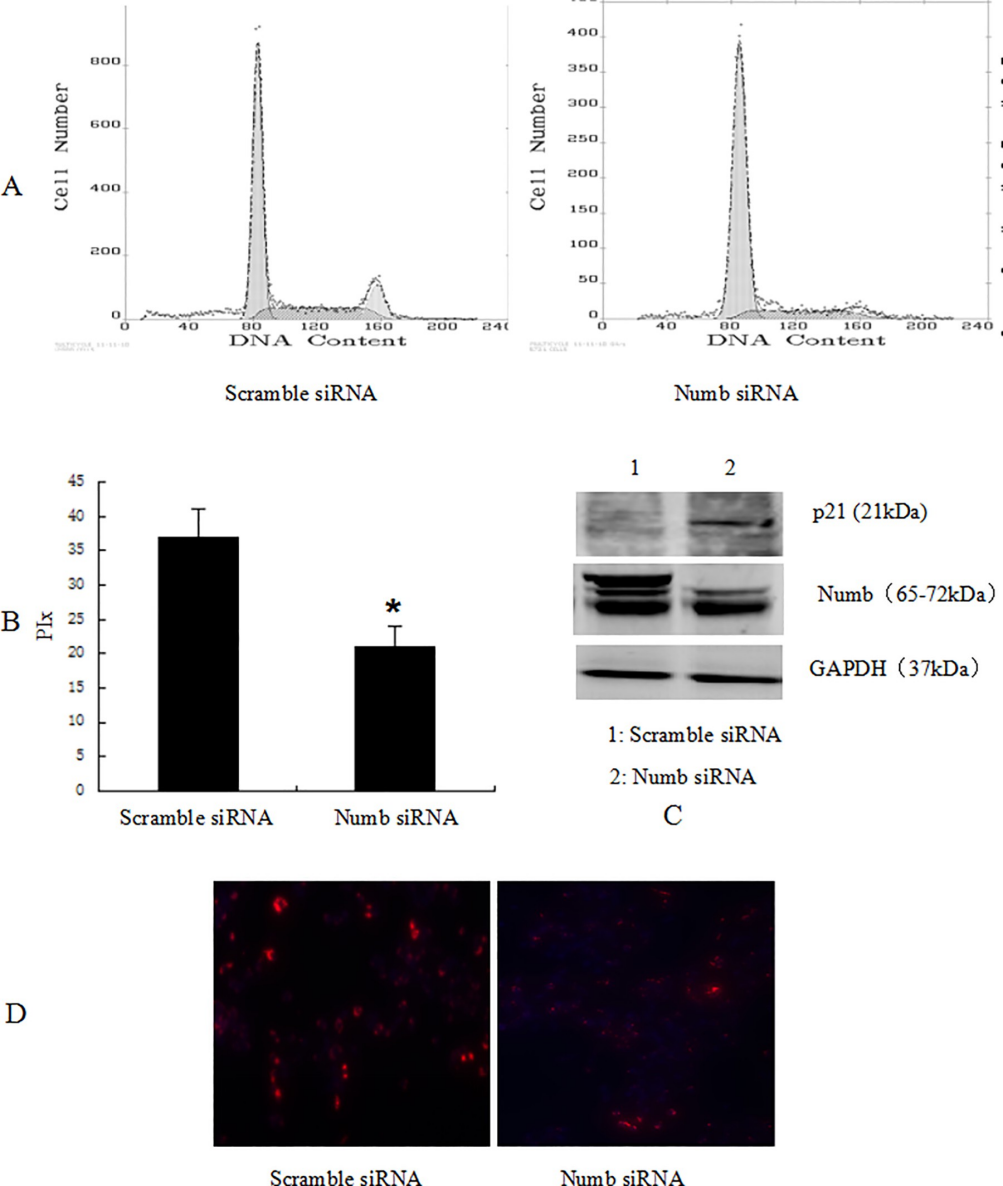
Knockdown of Numb up-regulated the expression of p21, inhibited BEL-7402 cell cycle progression. BEL-7402 cells were transfected with scramble siRNA, Numb siRNA, respectively. 48 h after transfection, the amount of Numb and p21 was determined by antibodies specific for Numb and p21(C). (A)Cell cycle was analysed by FACSCalibur (Becton Dickinson, Mountain View, CA). The proliferative index (PIx) decreased compared to control (P<0.05, B) For BrdU incorporation analysis (D), the cells were incubated with a mouse monoclonal anti-BrdU antibody overnight at 4°C, and were incubated with fluorescein isothiocyanate-conjugated goat antimouse IgG for 1 h at room temperature. Hoechst 33342 was used to label nuclei (red). Photographs were taken using a Nikon microscope. Magnification100x. The proliferation cells (red) decreased significantly.

Data supporting Figs 2, 4, 6, 7, 8 are provided with this notice in S1–S5 Files.

The authors apologize for the errors in the published article.

## Supporting information

S1 File [Fig 2.zip] Underlying data to support Fig 2. (ZIP)

S2 File [Fig 4.zip] Underlying data to support Fig 4. (ZIP)

S3 File [Fig 6.zip] Underlying data to support Fig 6. (ZIP)

S4 File [Fig 7.zip] Underlying data to support Fig 7. (ZIP)

S5 File [Fig 8.zip] Underlying data to support Fig 8. (ZIP)
