# Supplementary figures and images for: Correction: Numb Promotes Cell Proliferation and Correlates with Poor Prognosis in Hepatocellular Carcinoma
Source: PLoS One. 2022 Mar 23;17(3):e0265938. doi: 10.1371/journal.pone.0265938 (PMC8942234; doi:10.1371/journal.pone.0265938)

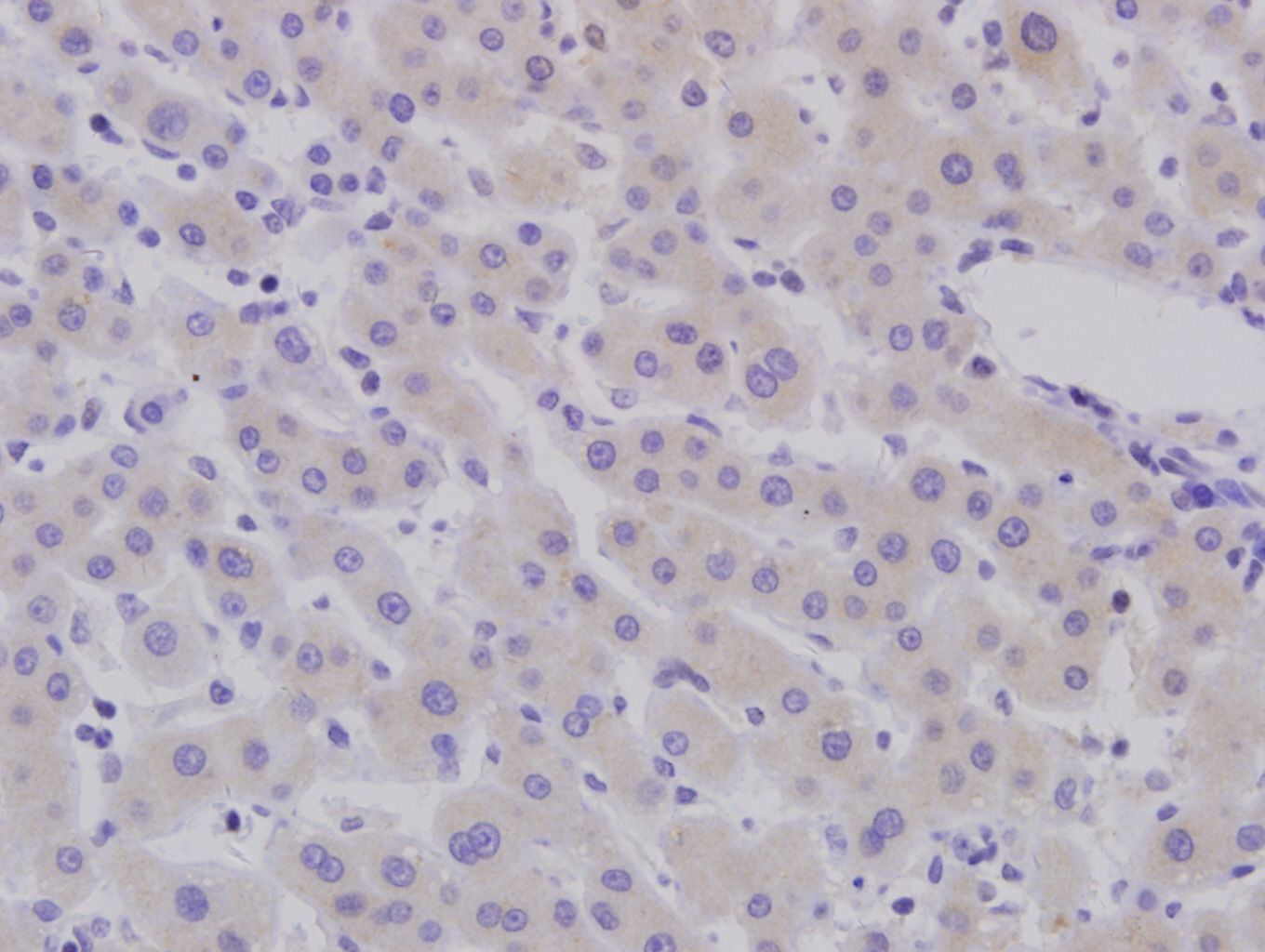

Supplement: S1 File — (ZIP) [file pone.0265938.s001.zip › patient 1/1.N.jpg]

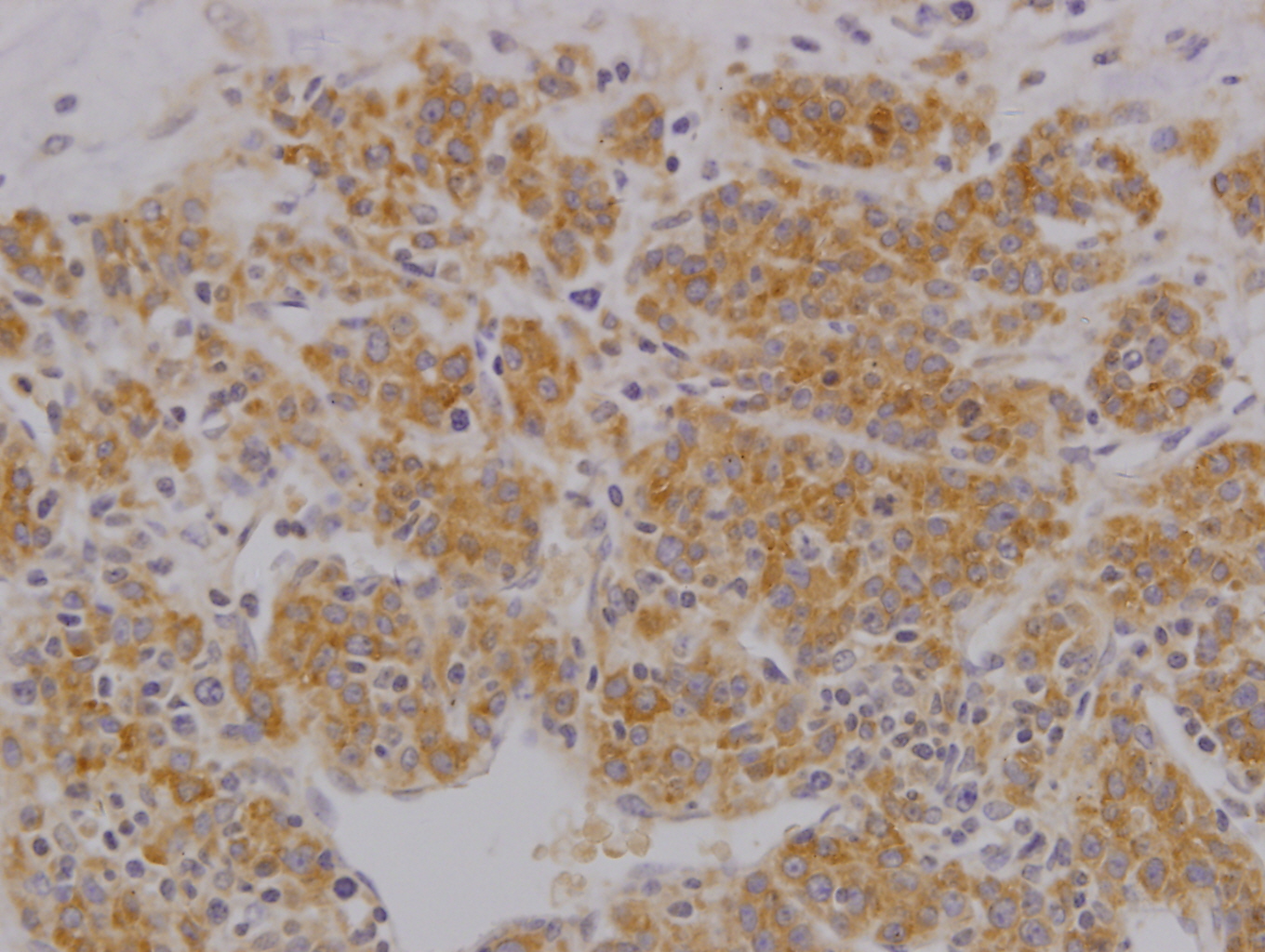

Supplement: S1 File — (ZIP) [file pone.0265938.s001.zip › patient 1/1.T.jpg]

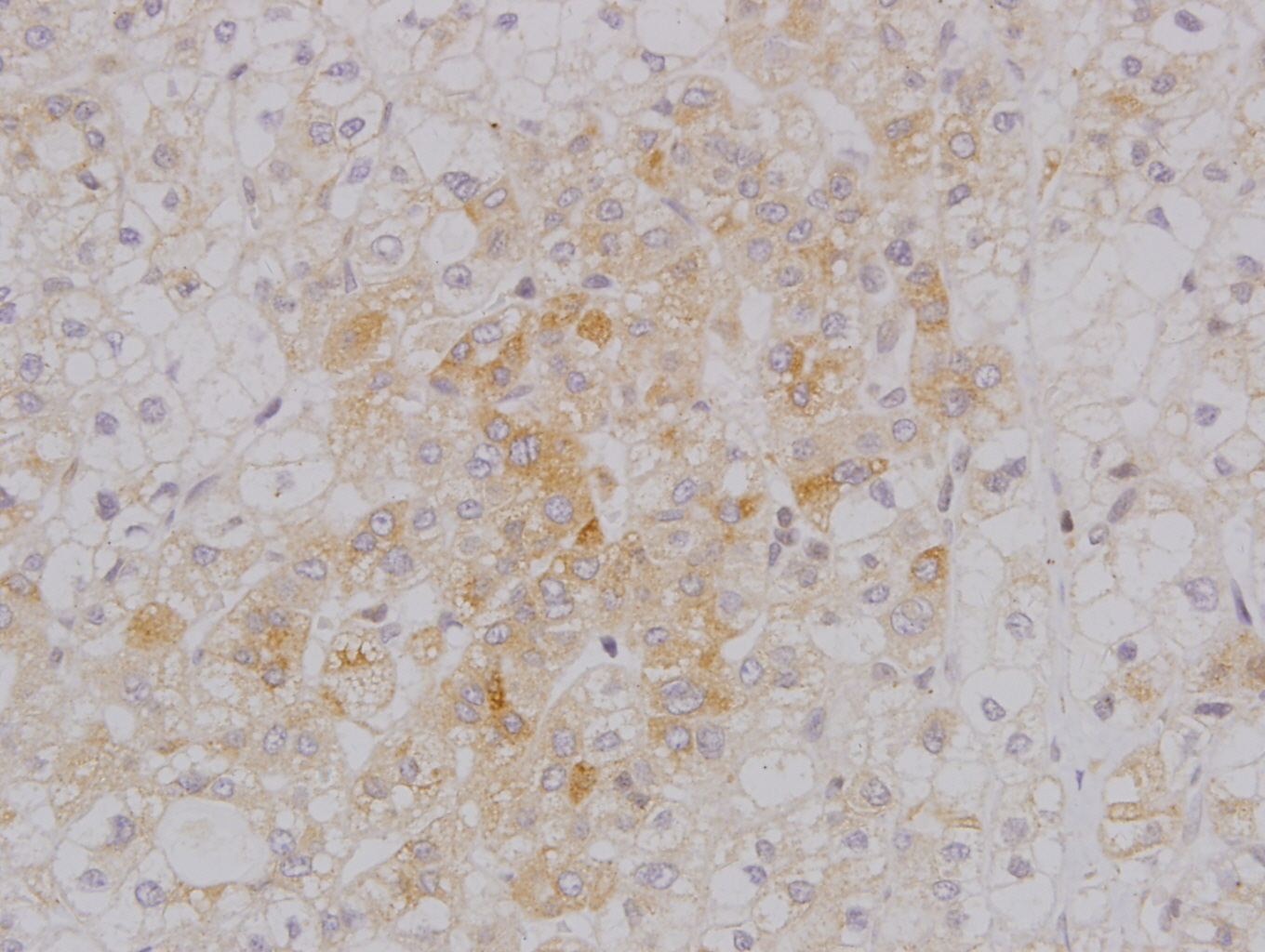

Supplement: S1 File — (ZIP) [file pone.0265938.s001.zip › patient 2/2.N.jpg]

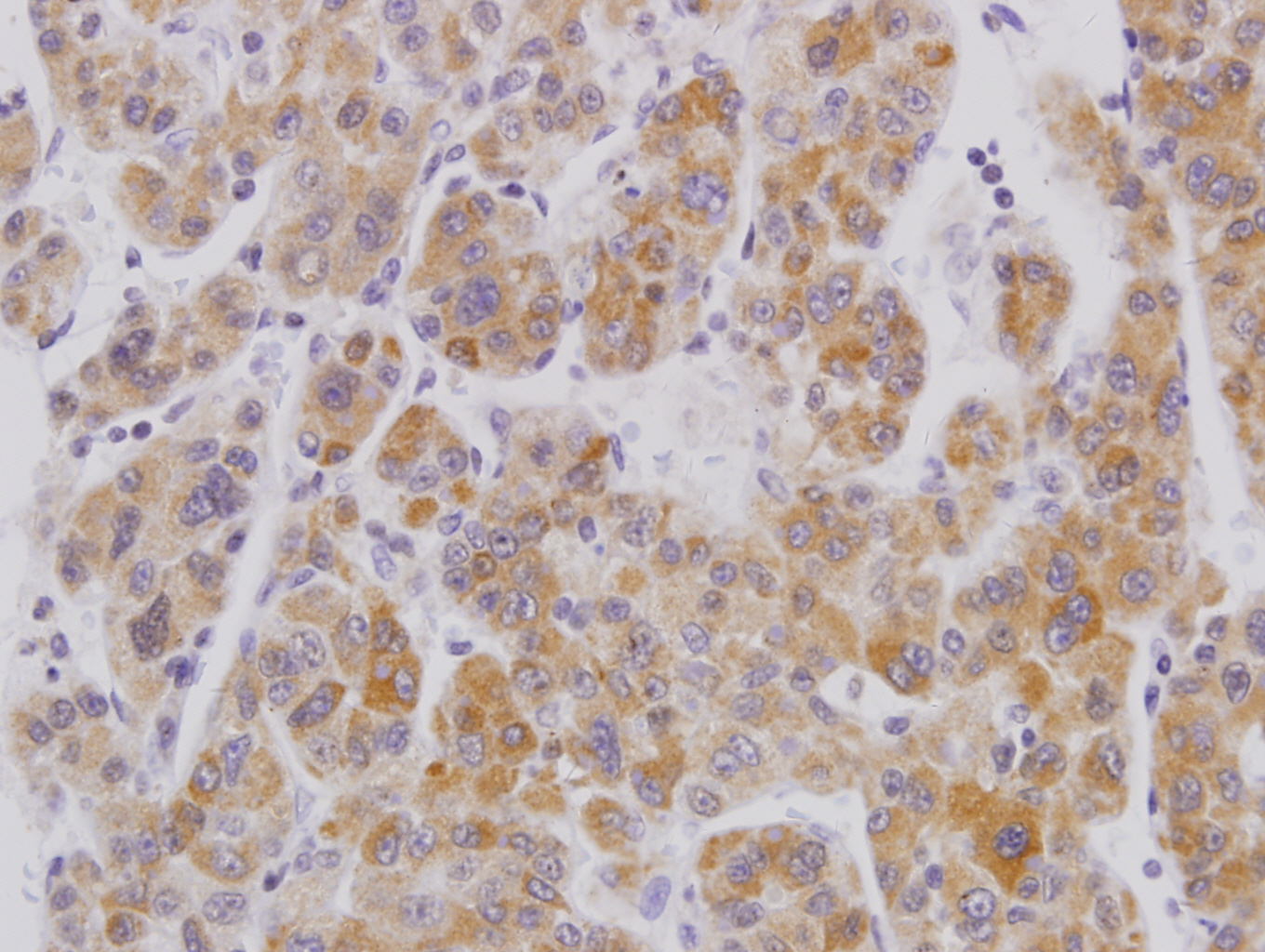

Supplement: S1 File — (ZIP) [file pone.0265938.s001.zip › patient 2/2.T.jpg]

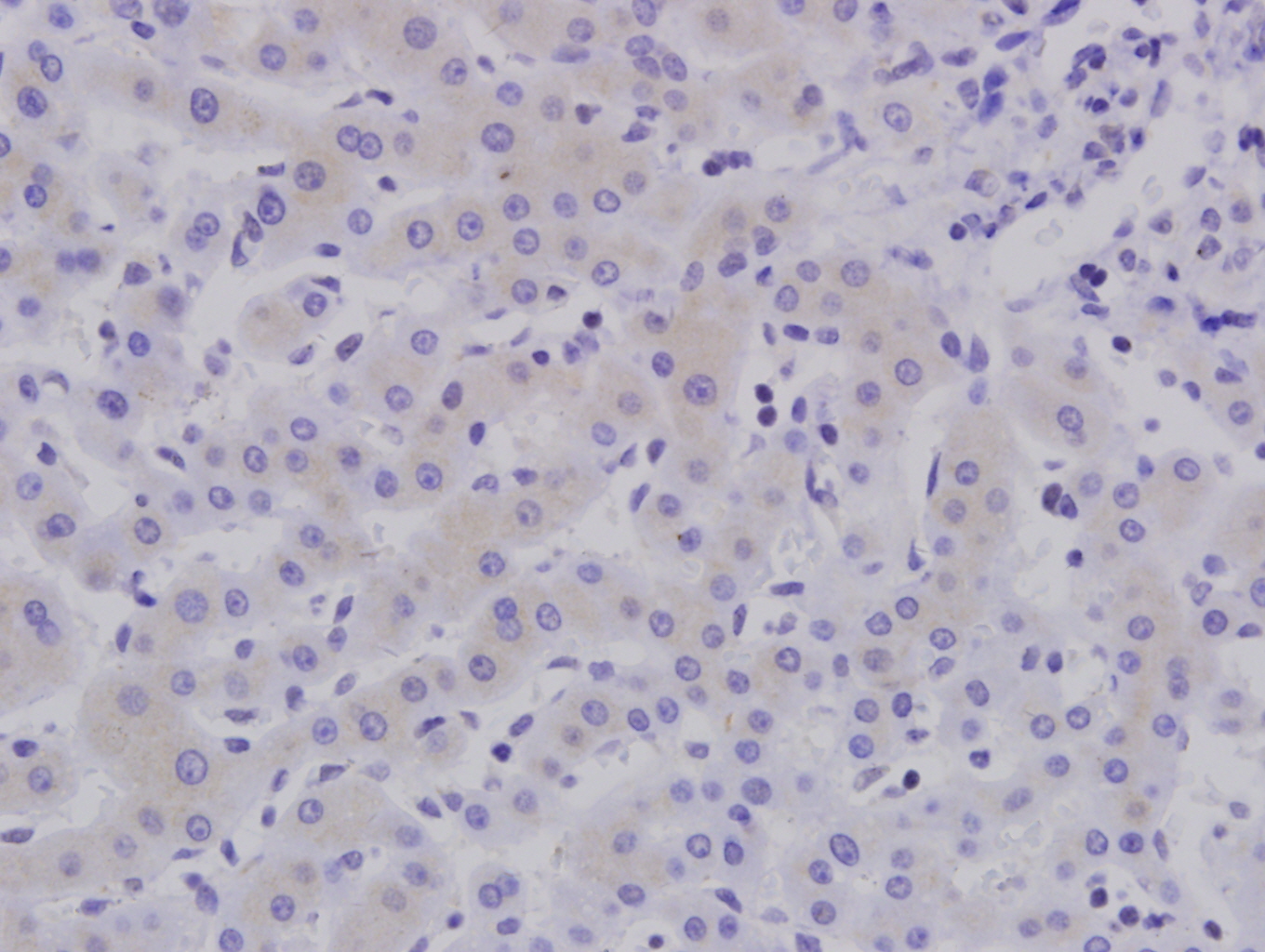

Supplement: S1 File — (ZIP) [file pone.0265938.s001.zip › patient 3/3.N.jpg]

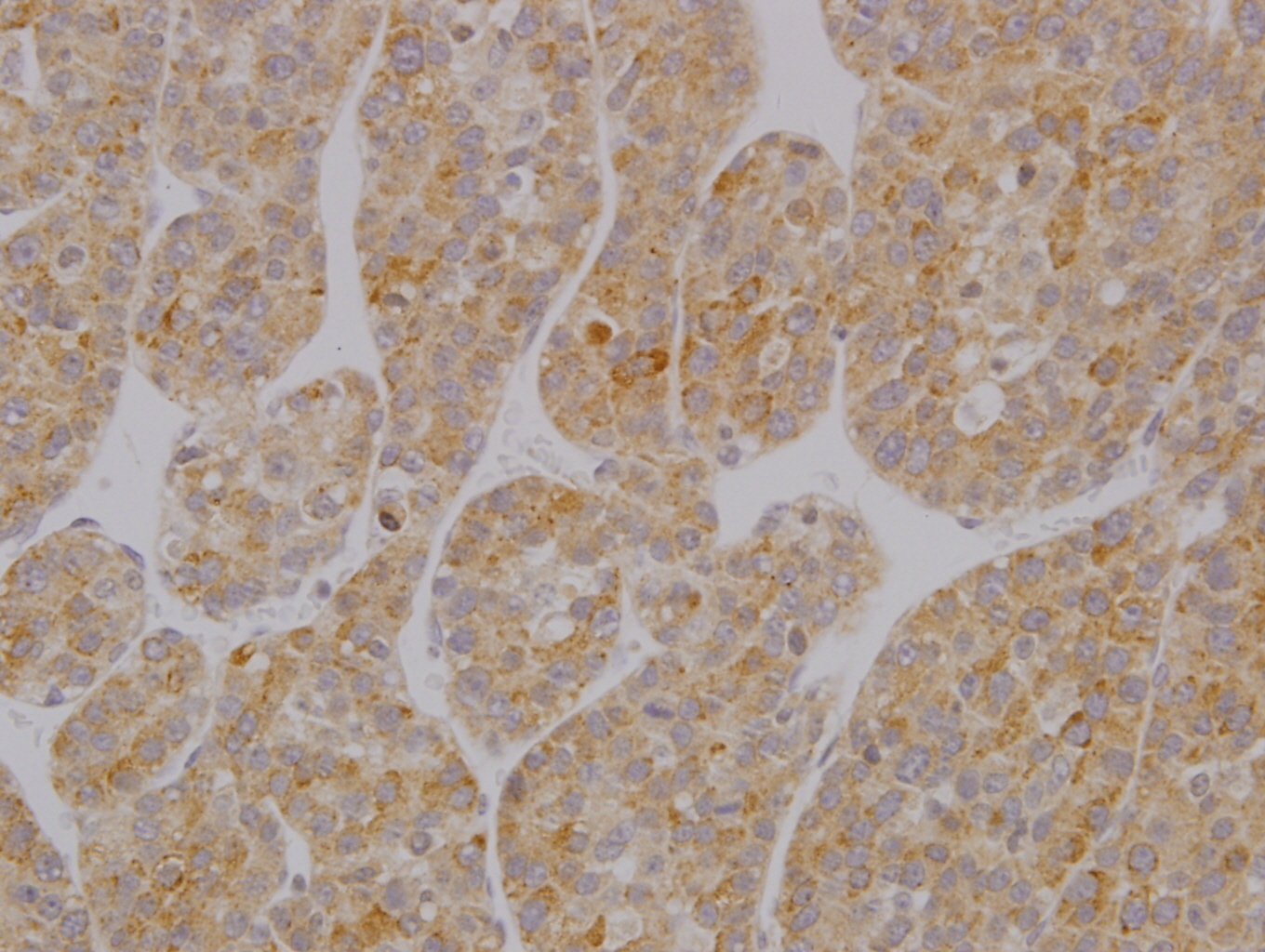

Supplement: S1 File — (ZIP) [file pone.0265938.s001.zip › patient 3/3.T.jpg]

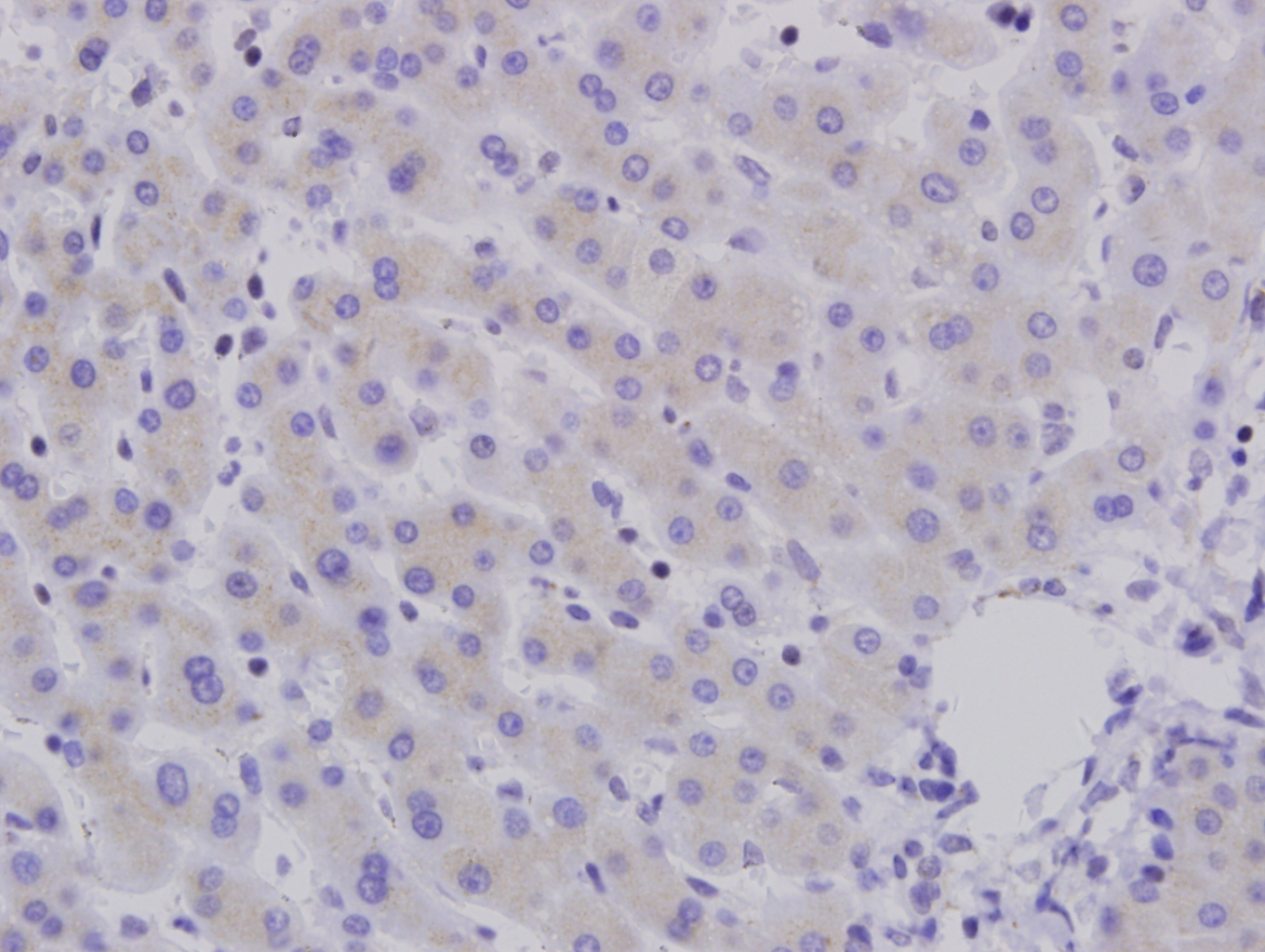

Supplement: S1 File — (ZIP) [file pone.0265938.s001.zip › patient 4/4.N.jpg]

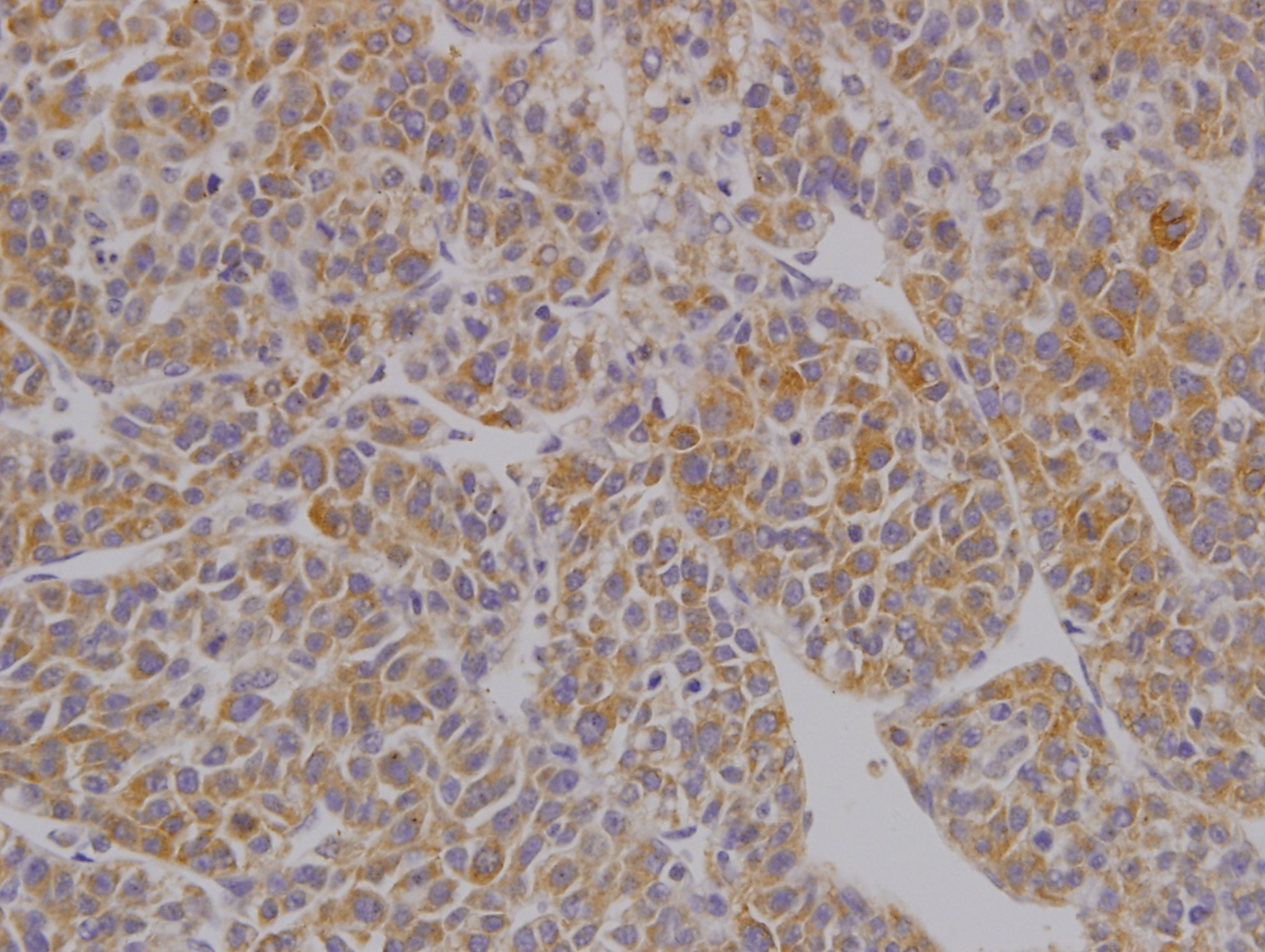

Supplement: S1 File — (ZIP) [file pone.0265938.s001.zip › patient 4/4.T.jpg]

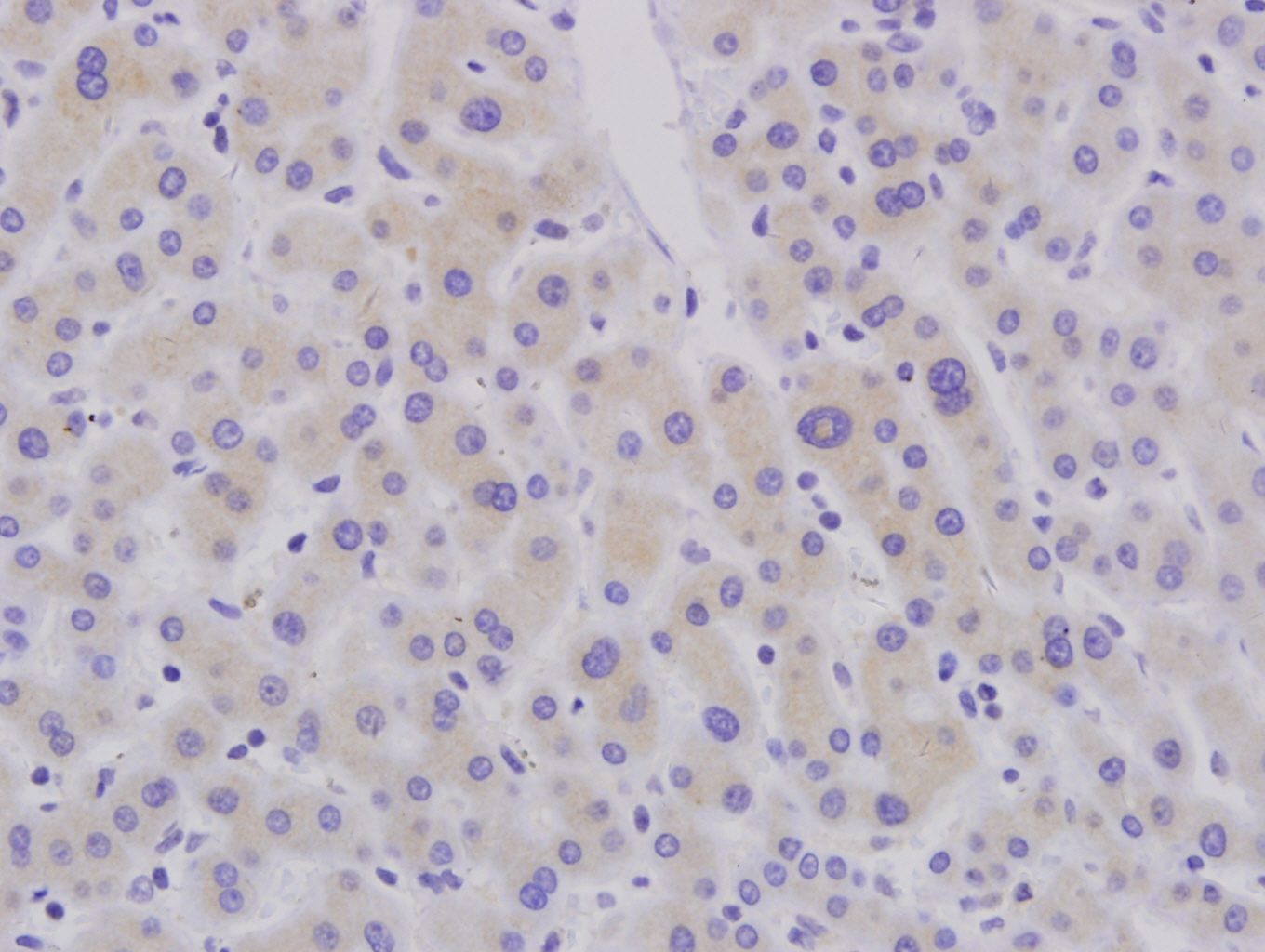

Supplement: S1 File — (ZIP) [file pone.0265938.s001.zip › patient 5/5.N.jpg]

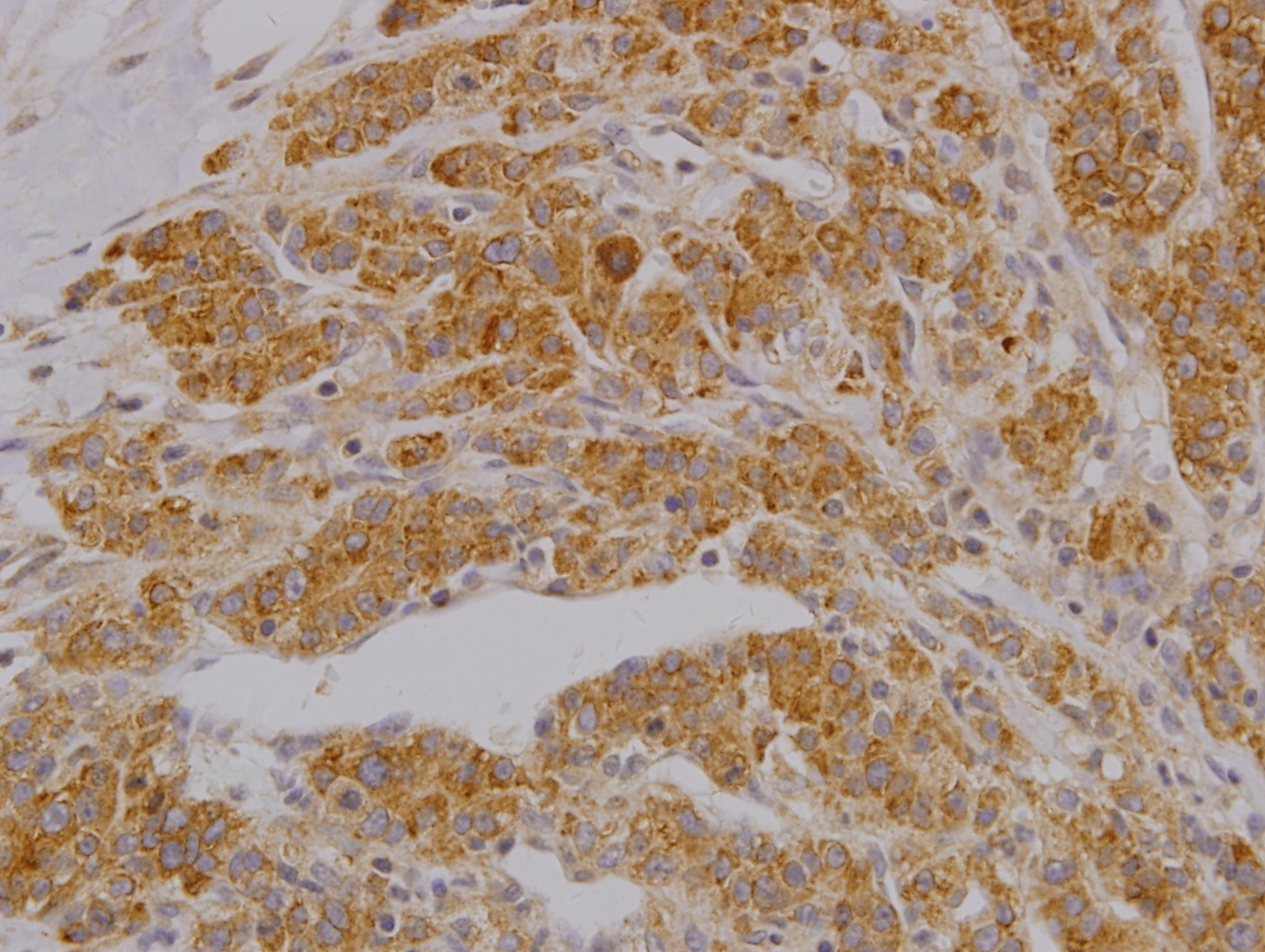

Supplement: S1 File — (ZIP) [file pone.0265938.s001.zip › patient 5/5.T.jpg]

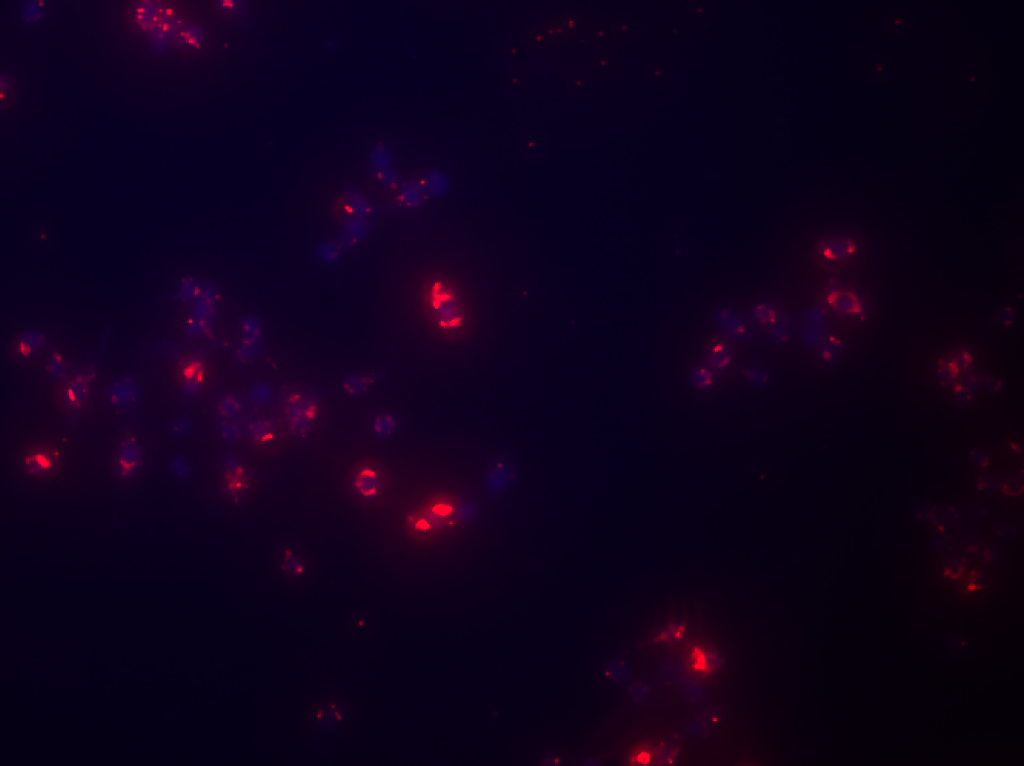

Supplement: S2 File — (ZIP) [file pone.0265938.s002.zip › 4 D Numb.tif]

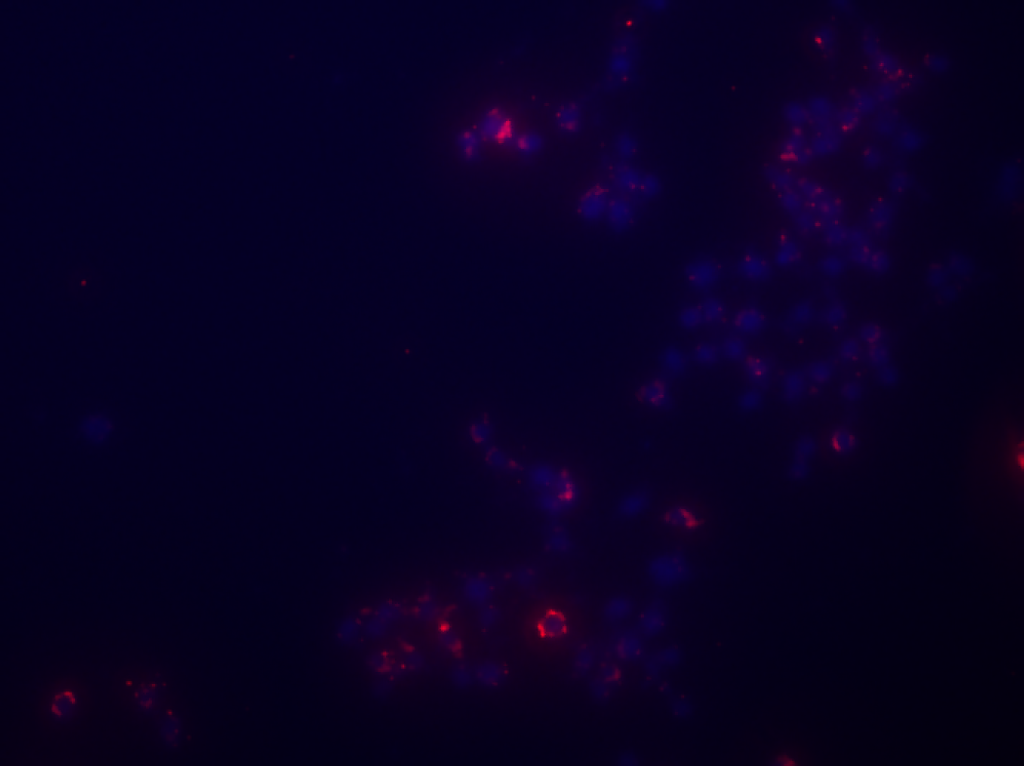

Supplement: S2 File — (ZIP) [file pone.0265938.s002.zip › 4 D vector.tif]

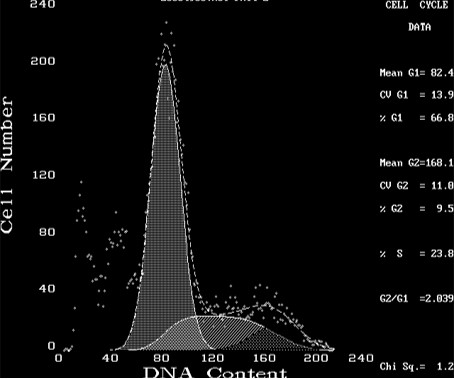

Supplement: S2 File — (ZIP) [file pone.0265938.s002.zip › Numb 1.jpg]

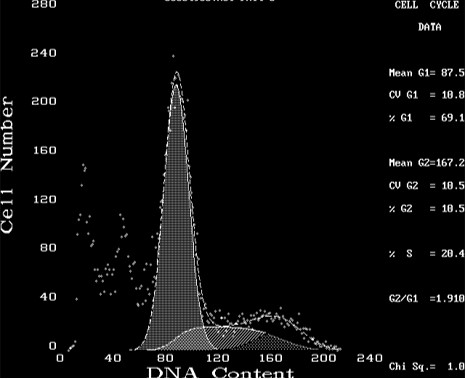

Supplement: S2 File — (ZIP) [file pone.0265938.s002.zip › Numb 2.jpg]

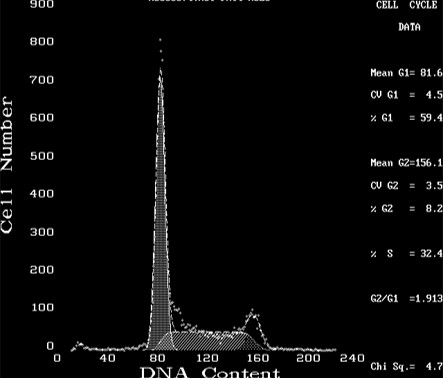

Supplement: S2 File — (ZIP) [file pone.0265938.s002.zip › Numb 3.jpg]

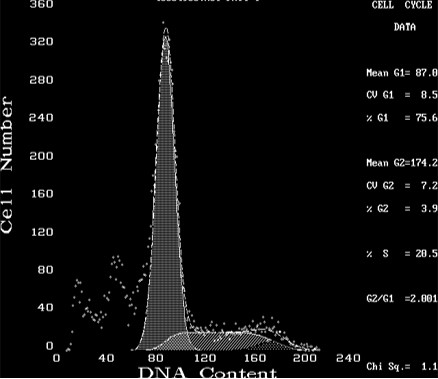

Supplement: S2 File — (ZIP) [file pone.0265938.s002.zip › vector 1.jpg]

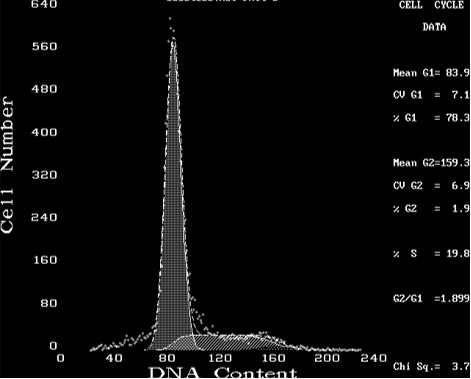

Supplement: S2 File — (ZIP) [file pone.0265938.s002.zip › Vector 2.jpg]

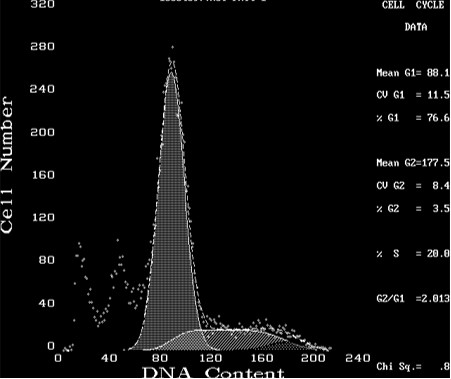

Supplement: S2 File — (ZIP) [file pone.0265938.s002.zip › vector 3.jpg]

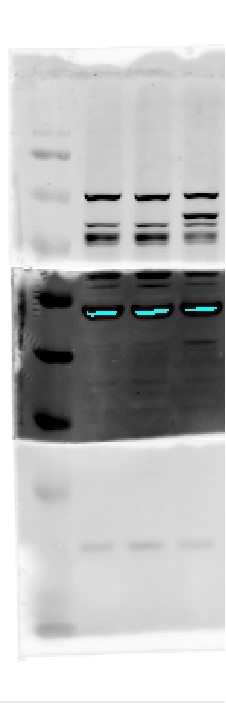

Supplement: S2 File — (ZIP) [file pone.0265938.s002.zip › WB.jpg]

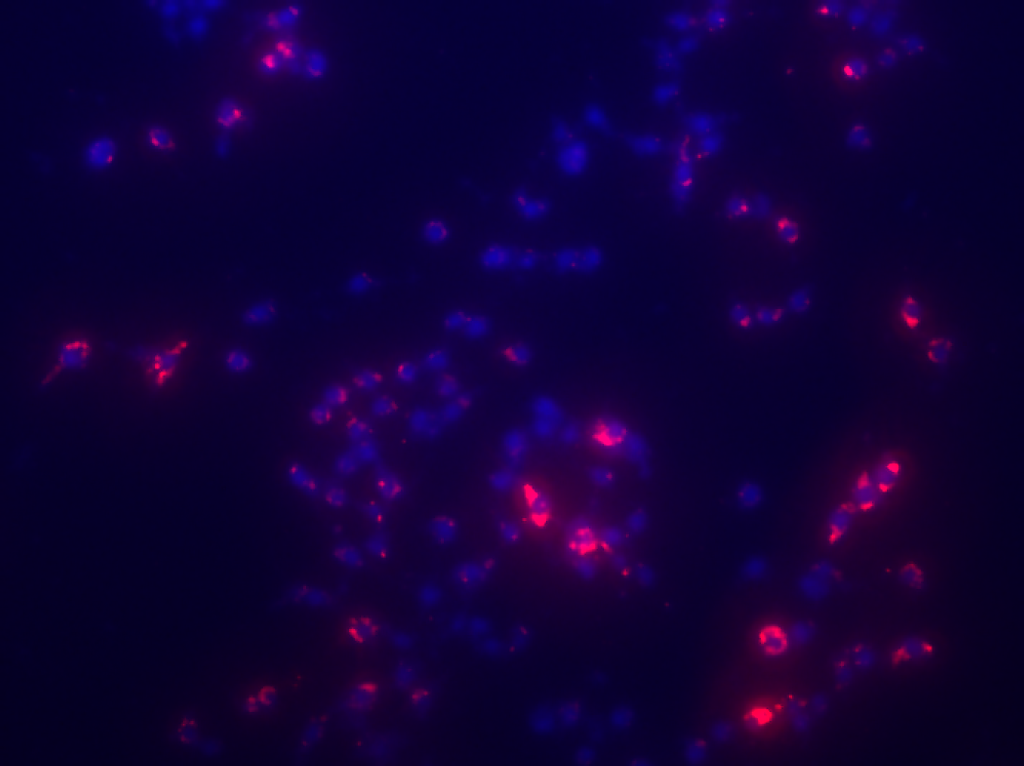

Supplement: S3 File — (ZIP) [file pone.0265938.s003.zip › 6 D NumbsiRNA.tif]

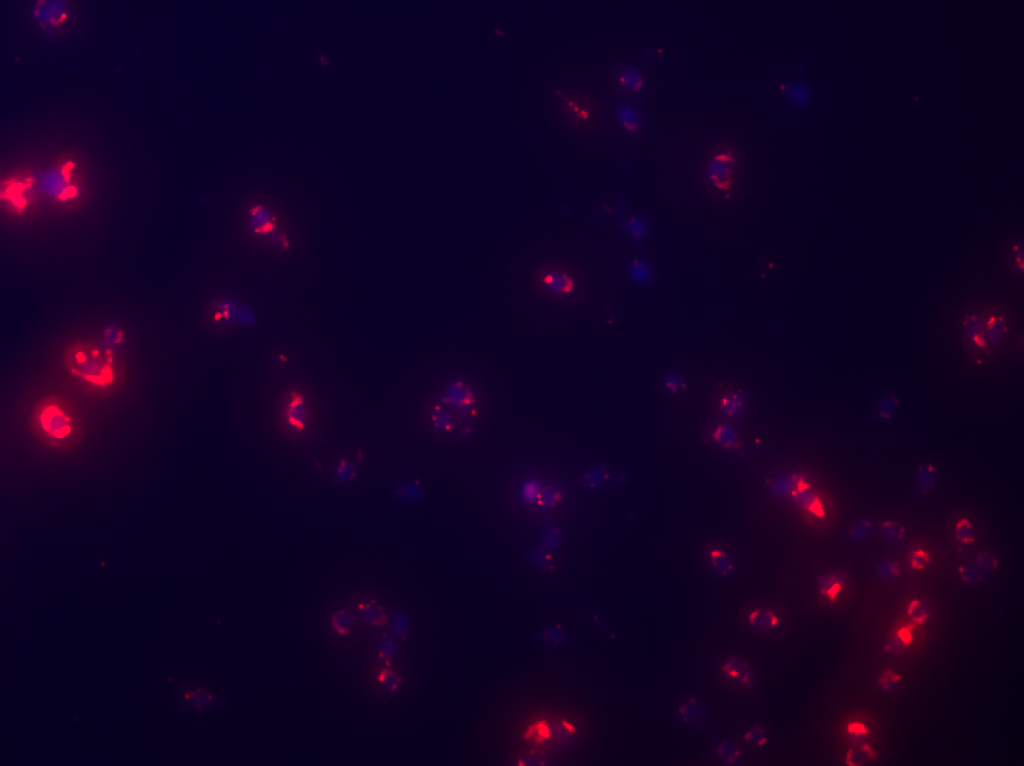

Supplement: S3 File — (ZIP) [file pone.0265938.s003.zip › 6 D Scramble siRNA .tif]

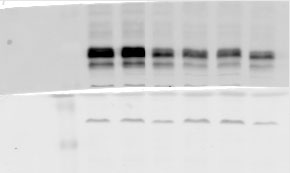

Supplement: S3 File — (ZIP) [file pone.0265938.s003.zip › Numb and GAPDH.jpg]

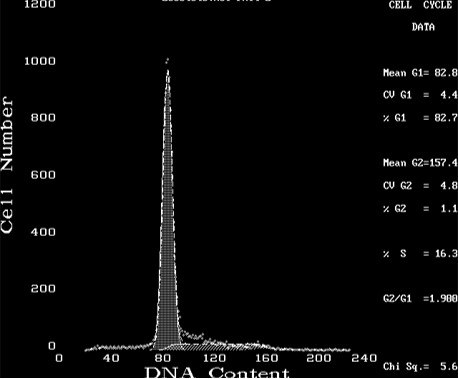

Supplement: S3 File — (ZIP) [file pone.0265938.s003.zip › NumbsiRNA 1.jpg]

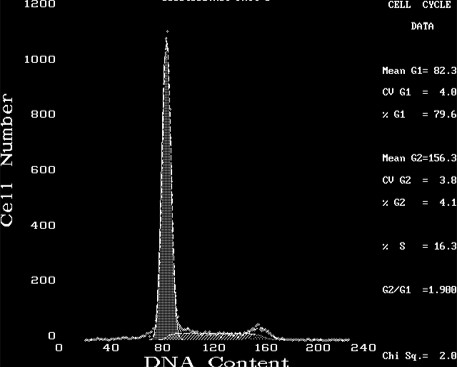

Supplement: S3 File — (ZIP) [file pone.0265938.s003.zip › NumbsiRNA 2.jpg]

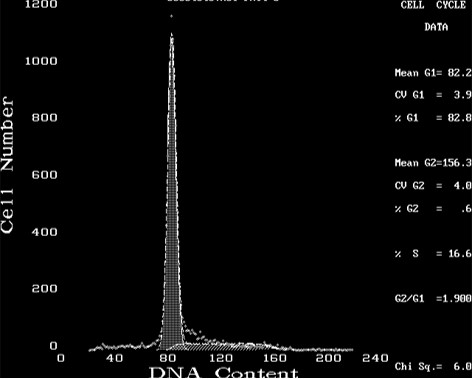

Supplement: S3 File — (ZIP) [file pone.0265938.s003.zip › NumbsiRNA 3.jpg]

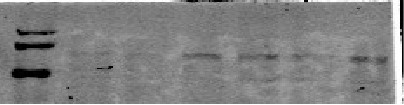

Supplement: S3 File — (ZIP) [file pone.0265938.s003.zip › P21.jpg]

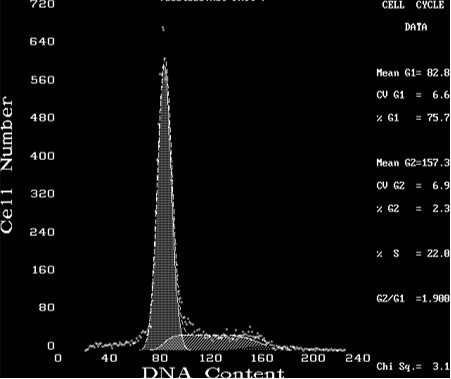

Supplement: S3 File — (ZIP) [file pone.0265938.s003.zip › Scramble siRNA 1.jpg]

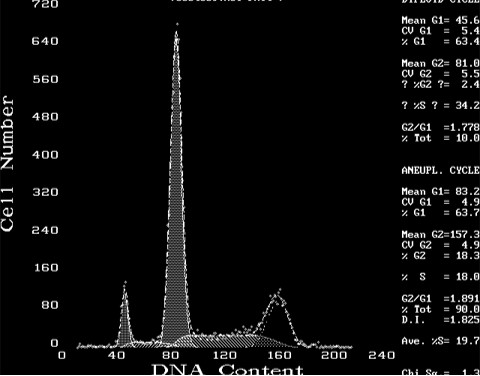

Supplement: S3 File — (ZIP) [file pone.0265938.s003.zip › Scramble siRNA 2.jpg]

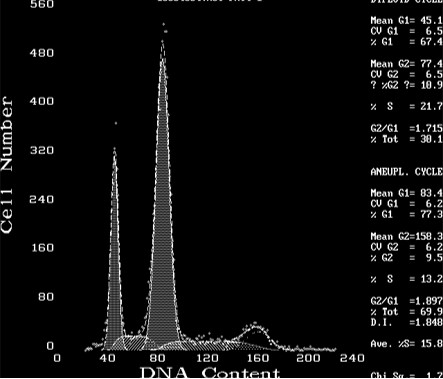

Supplement: S3 File — (ZIP) [file pone.0265938.s003.zip › Scramble siRNA 3.jpg]

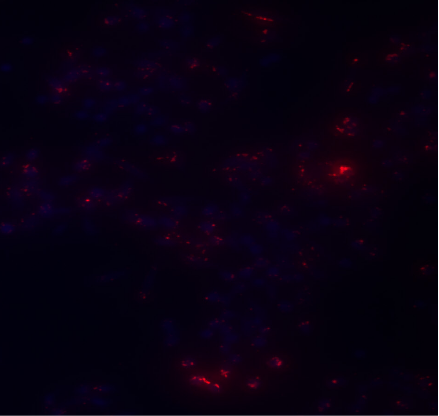

Supplement: S4 File — (ZIP) [file pone.0265938.s004.zip › 07 D Numb siRNA.tif]

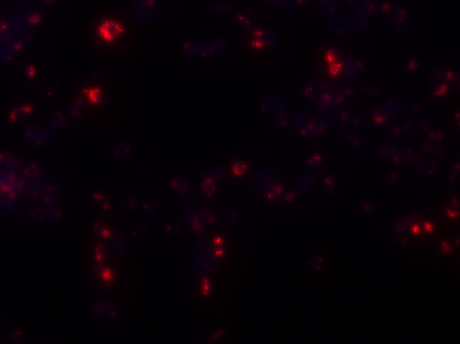

Supplement: S4 File — (ZIP) [file pone.0265938.s004.zip › 07 D Scramble siRNA.tif]

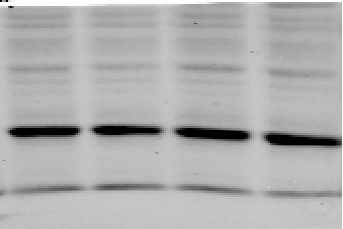

Supplement: S4 File — (ZIP) [file pone.0265938.s004.zip › GAPDH.jpg]

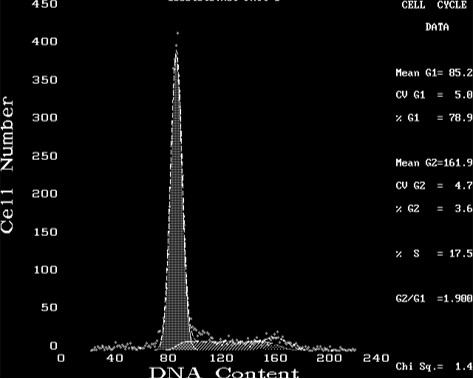

Supplement: S4 File — (ZIP) [file pone.0265938.s004.zip › Numb siRNA 1.jpg]

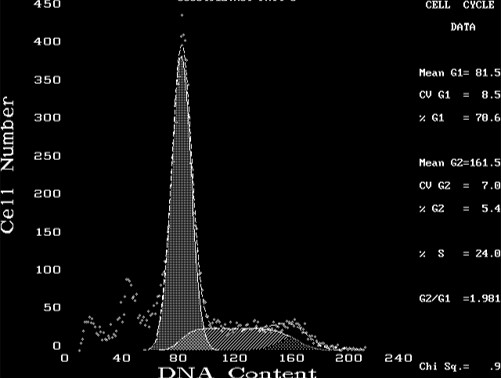

Supplement: S4 File — (ZIP) [file pone.0265938.s004.zip › Numb siRNA 2.jpg]

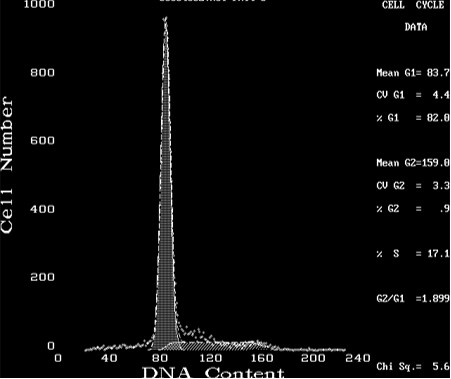

Supplement: S4 File — (ZIP) [file pone.0265938.s004.zip › Numb siRNA 3.jpg]

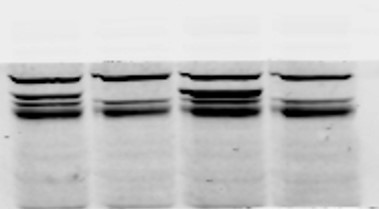

Supplement: S4 File — (ZIP) [file pone.0265938.s004.zip › Numb.jpg]

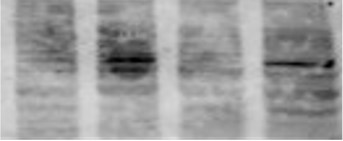

Supplement: S4 File — (ZIP) [file pone.0265938.s004.zip › P21.jpg]

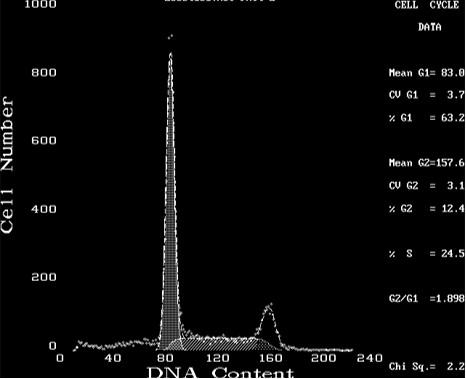

Supplement: S4 File — (ZIP) [file pone.0265938.s004.zip › Scramble siRNA 1.jpg]

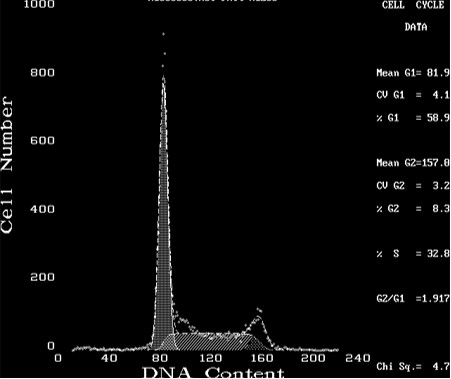

Supplement: S4 File — (ZIP) [file pone.0265938.s004.zip › Scramble siRNA 2.jpg]

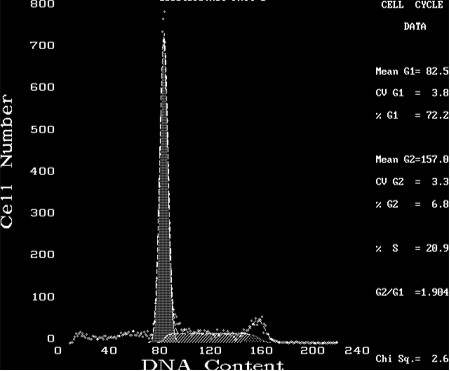

Supplement: S4 File — (ZIP) [file pone.0265938.s004.zip › Scramble siRNA 3.jpg]

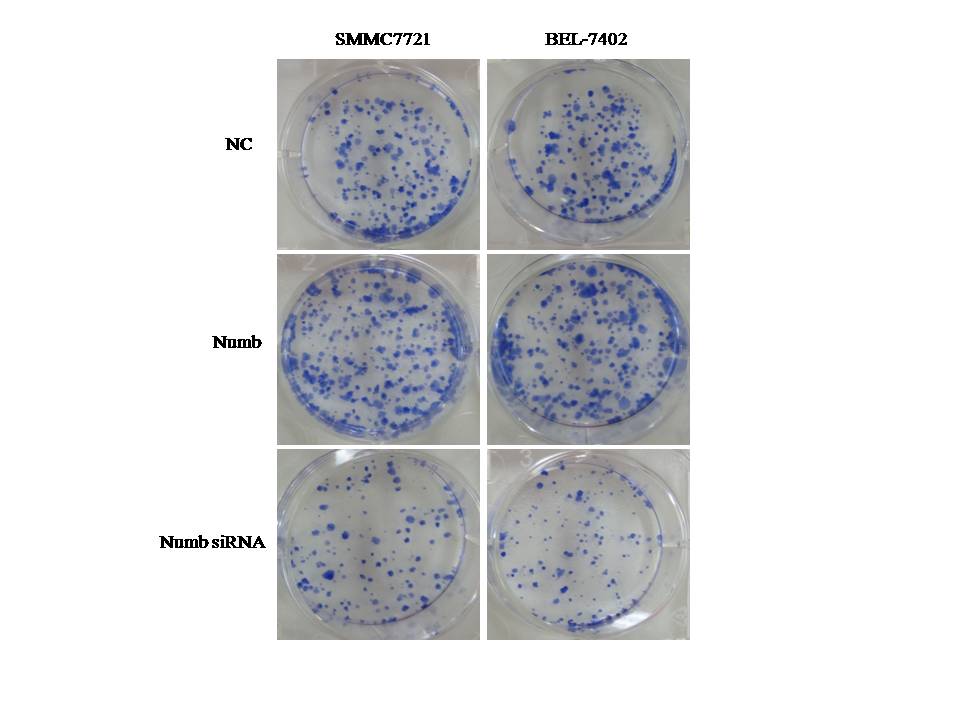

Supplement: S5 File — (ZIP) [file pone.0265938.s005.zip › Figure8.1.JPG]

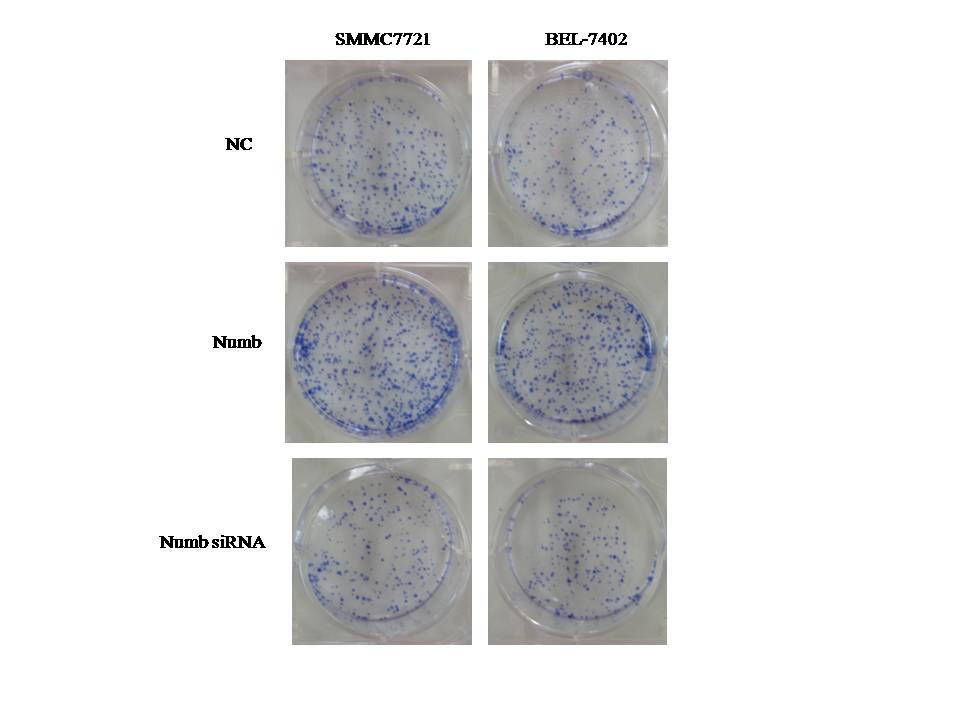

Supplement: S5 File — (ZIP) [file pone.0265938.s005.zip › Figure8.2.JPG]

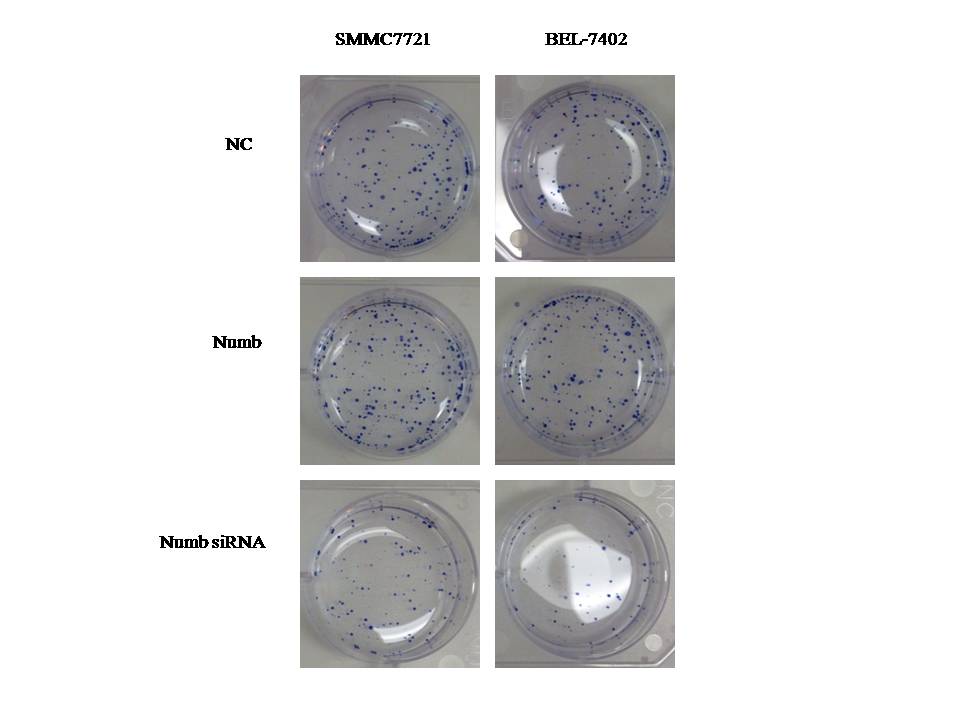

Supplement: S5 File — (ZIP) [file pone.0265938.s005.zip › Figure8.3.JPG]
